# The impact of mental health and psychosocial support programmes on children and young people’s mental health in the context of humanitarian emergencies in low- and middle-income countries: A systematic review and meta-analysis

**DOI:** 10.1017/gmh.2024.17

**Published:** 2024-02-12

**Authors:** Mukdarut Bangpan, Lambert Felix, Farida Soliman, Preethy D’Souza, Anna-Theresa Jieman, Kelly Dickson

**Affiliations:** 1 The Evidence for Policy and Practice information and Co-ordinating Centre (EPPI-Centre), UCL Social Research Institute, University College London, London, United Kingdom; 2 School of Primary, Community and Social Care, Keele University, Keele, Staffordshire, United Kingdom; 3 Linguistics Department, Queen Mary University of London, London, United Kingdom; 4 Department of Biological and Experimental Psychology, School of Biological and Behavioural Sciences, Queen Mary University of London, London, United Kingdom

**Keywords:** Systematic review, Mental Health, Humanitarian Emergencies, Children, Young people

## Abstract

Humanitarian emergencies pose a significant global health challenge for children and young people’s mental and psychological health. This systematic review investigates the effectiveness of mental health and psychosocial support (MHPSS) programmes delivered to children and young people affected by humanitarian emergencies in low- and middle-income countries (LMICs). Twelve electronic databases, key websites and citation checking were undertaken. Forty-three randomised controlled trials (RCTs) published in English between January 1980 and May 2023 were included in the review. Overall, the findings suggest that cognitive behavioural therapy may improve depression symptoms in children and young people affected by humanitarian emergencies. Narrative exposure therapy may reduce feelings of guilt. However, the impact of the other MHPSS modalities across outcomes is inconsistent. In some contexts, providing psychosocial programmes involving creative activities may increase the symptoms of depression in children and young people. These findings emphasise the need for the development of MHPSS programmes that can safely and effectively address the diverse needs of children and young people living in adversarial environments.

## Impact statements

Mental health and Humanitarian Emergencies are global issues, affecting over 200 million school-aged children and young people, placing them at risk of developing mental health conditions (Wait, 2022; UNICEF, 2023). Mental Health and Psychosocial Support (MHPSS) programmes, aiming to protect, promote, prevent and/or treat mental health conditions, are considered as a key priority by international actors in humanitarian emergencies. In recent years, there have been growing interests to explore the impact of MHPSS on children and young people. Contributing to this effort, we identified 43 randomised controlled trials evaluating different types and modalities of MHPSS on a wide range of outcomes. Overall, MHPSS programmes that explicitly link thoughts, emotions, feelings and behaviour, such as cognitive behavioural programmes, can potentially improve depression symptoms. Additionally, narrative exposure therapy may contribute to the reduction in feelings of guilt. Psychosocial programmes involving creative activities indicate potential unintended effects on depression. However, evidence regarding the impact of other MHPSS modalities remains inconclusive. The lack of clear and consistent findings across MHPSS modalities underscores the importance of gaining an understanding how implementation contexts and socio-cultural dimensions may, directly and indirectly, impact children and young people’s mental health and well-being. This systematic review highlights the importance of tailoring MHPSS programmes to diverse needs in order to safely and effectively promote, prevent and treat mental health conditions in children and young people affected by humanitarian crises in low- and middle-income countries.

## Introduction

In recent years, millions of children and young people worldwide have been affected by extreme weather episodes, migration, conflicts, forced displacement and global public health emergencies, including COVID-19 (UNICEF, 2021). Many of them are displaced from their homes and separated from their parents and guardians, at risk of being recruited into their national armed forces, or exposed to adverse childhood experiences, including violence, serious physical injuries and extreme poverty (Bennouna et al., 2020; Ceccarelli et al., 2022). Repeated, sudden or prolonged exposures to these traumatic events can have a severe and long-term impact on their mental health and well-being (Miller and Jordans, 2016; Ataullahjan et al., 2020). Children and young people living in low- and middle-income countries (LMICs) often are less prepared and have limited access to basic services and essential resources to respond to humanitarian emergencies.

Mental health and psychosocial support (MHPSS) is increasingly considered an essential element of humanitarian responses to support children and young people affected by humanitarian crises in LMICs (Meyer and Morand, 2015). International organisations, such as UNICEF, consider MHPSS as a priority, aiming to enhance the implementation of MHPSS across humanitarian sectors (UNICEF, 2019). Similarly, the WHO-funded Inter-Agency Standing Committee (IASC) has introduced guidelines for MHPSS implementation in emergencies, providing a framework to understand different layers of programming and valuable activities to facilitate the development of effective, evidence-based MHPSS across agencies and practices (IASC, 2007b).

Several systematic reviews have explored the impact of MHPSS on mental health outcomes in children and young people affected by humanitarian crises (Jordans et al., 2009; Tol et al., 2013; Jordans et al., 2016; Brown et al., 2017; Morina et al., 2017; Bosqui and Marshoud, 2018; Purgato et al., 2018b; Pedersen et al., 2019; Barbui et al., 2020; Kamali et al., 2020; Papola et al., 2020; Pfefferbaum et al., 2020; Purgato et al., 2020; Uppendahl et al., 2020; Galvan et al., 2021). In general, most reviews indicate a positive impact of psychological and psychosocial interventions on post-traumatic symptoms in children and young people. However, the effect of MHPSS on internalised symptoms such as depression and anxiety remains uncertain. Purgato and colleagues (2018), in their individual patient data meta-analysis, observed no impact of the focused psychosocial interventions on depression and anxiety. In contrast, the Uppendahl study (2020) suggested that psychological and psychosocial interventions had a positive effect on the combined outcomes of post-traumatic stress disorders (PTSD), depression and anxiety. Moreover, MHPSS programmes evaluated in previous reviews were tailored to the varied needs of children and young people in humanitarian contexts. This complex nature of MHPSS programming poses a challenge in identifying effective modalities. Recent evidence reviews on health interventions during humanitarian crises have recommended future research to enhance understanding of the effectiveness and implementation of different MHPSS modalities for diverse populations including children (Barbui et al., 2020; Doocy et al., 2022). Given the considerable number of children and young people in need of MHPSS in humanitarian emergencies in LMICs, this systematic review is timely. It builds on existing literature, by systematically describing the current research landscape and the nature of existing MHPSS modalities evaluated and delivered to children and young people affected by humanitarian emergencies in LMICs. We also examine the effects and potential adverse consequences to inform policy and practice in LMICs.

## Methods

### Search strategy and selection criteria

We carried out a systematic review of research evidence following PRISMA guidelines (Page et al., 2021). We searched 12 bibliographic databases across disciplines and specialist databases: Medline, ERIC, PsycINFO, Econlit, Cochrane Library, IDEAS, IBSS, CINHAL, Scopus, ASSIA, Web of Science and Sociological Abstracts. Both published and unpublished studies were comprehensively searched from the websites of relevant organisations. We searched the citations of included studies and relevant systematic reviews. Search strategies were informed by the scoping exercise (Bangpan et al., 2016) and were developed based on three key concepts (mental health and psychosocial support, humanitarian emergencies and study designs). The search was first performed in November 2015 and updated and finalised in May 2023 (see S1 for the example of database search strategies and a list of websites searched). We included studies that aimed to evaluate the impact of MHPSS programmes on mental health and well-being of children and young people aged at or below 25, who were affected by humanitarian emergencies in LMICs^1^ MHPSS programmes were broadly defined as interventions seeking to ‘*provide or promote psychosocial well-being and/or prevent or treat mental health disorder’* p. 1, (IASC, 2007b). We included only experimental studies with control groups that were published in English in or after 1980 (see S2 for eligibility criteria). Two reviewers (MB and KD) piloted the eligibility criteria. A pilot screening exercise was performed by the review team members (MB, LF, KD, FS, ZD, AJ) before independently screening the studies on titles and abstracts. Any discrepancies identified during both the pilot and independent screening were addressed through discussions between the reviewers. When there was insufficient information, full reports were obtained to assess the eligibility for inclusion.

The data extraction tool, developed and piloted by two reviewers (MB, KD), aimed to collect information on key characteristics of MHPSS programmes, implementation strategies, study design, findings and conclusions. Three reviewers (MB, FS, PD) independently extracted information from eligible studies, and the second reviewer carried out a consistency check of all included studies using EPPI-Reviewer (Thomas et al., 2020). Four reviewers (MB, FS, PD, LF) assessed the risk of bias of the studies included in the synthesis using the Cochrane risk-of-bias tool for randomised controlled trials (RCTs) (RoB2) (Sterne et al., 2019) and cluster randomised controlled trials (cRCTs) (RoB 2 for cRCTs) (Eldridge et al., 2017). The overall quality of the included studies was subsequently judged as a high risk of bias, some concerns or a low risk of bias according to RoB 2 framework. We resolved any disagreements by discussing and consulting with a third review member when required. The review protocol was registered at PROSPERO database (CRD42016033578).

## Data analysis

We first narratively described the key characteristics of all included studies. We included only RCTs and cRCTs. We classified types of MHPSS into five broad domains, including cognitive behavioural therapy (CBT), narrative exposure therapy (NET), interpersonal and body psychotherapy modalities, psychosocial programmes and psychoeducation (see Table 1) (Bangpan et al., 2019). The iterative process of MHPSS programme classification was undertaken. We read the description of the MHPSS programmes described by the authors of the included studies and then matched the programme descriptions against pre-defined programme definitions developed by the current review team. When appropriate, the meta-analysis was performed on conceptually similar outcome measures reported in more than one study using a random effect model. The pooled standardised mean difference (SMD) effect sizes were estimated and presented in forest plots with a 95% confidence interval (CI). We extracted outcome data at the longest follow up timepoint. When we included cRCTs, we checked whether the outcome data had been adjusted for intra-cluster correlation (ICC). In cases where studies did not report ICC, we used the ICC data based on other included studies. We assessed the extent of heterogeneity using *I^2^* statistics to quantify the magnitude of statistical heterogeneity and tested the statistical significance of heterogeneity using Q statistics. We ran a meta-analysis using STATA version 17.

**Table 1.** Types of MHPSS programmes

Types	Components and characteristics
Cognitive Behavioural Therapy (CBT)	- Provide face-to-face, individual or group talking therapy - Explore and make an explicit link between specific thoughts, emotions, somatic and non-somatic feelings and behaviour; and/or - Seek to positively change a person’s thinking (‘cognitive’) to elicit change in what they do (‘behavioural’) This programme of grouping encompassed trauma-focused CBT and is considered a rapidly growing heterogeneous group of MHPSS programmes drawing on selective elements of CBT approaches such as transdiagnostic therapies or therapies focused on schema, cognitive processing or behavioural activation only.
Narrative Exposure Therapy (NET)	- Facilitate exposure to specific or non-specific reminders, cues or memories related to exposure to a traumatic event; and - Support a person to reconstruct a consistent and/or coherent narrative about their traumatic experience, either verbally or through writing, to aid symptom reduction
Interpersonal and body psychotherapy	- Provide interpersonal, 1:1, talking or body-focused psychotherapy; and - Address the intra-psychic (i.e., internal world of the individual) and/or interpersonal issues arising from exposure to, humanitarian crises to support improved overall psychological functioning and coping skills. The MHPSS programmes in this group adopted a range of different therapeutic approaches to address broader psychological concerns, such as questions of meaning (e.g., existential-oriented therapy), social connectedness and depression (interpersonal therapy), on both a verbal and non-verbal level (e.g., yoga, eye movement desensitisation and reprocessing)
Psychosocial programme	- Support individuals, families and communities by developing and building on existing coping mechanisms to manage the impact of humanitarian crises; and/or - Focus on understanding people’s experience of humanitarian crises within broader social dimensions to facilitate individual and community resilience strategies to mitigate the impact This group of interventions encompassed a broad range of programme components beyond ‘talking therapy’, such as peer-to-peer support or active and recreational activities. Programme components were conceptualised as key routes to enable people to build resilient life trajectories by strengthening social and mental competencies to support individuals to manage and adapt to the adversity of humanitarian crises more effectively.
Psychoeducation	- Solely provide education on the impact of exposure to humanitarian crises; and/or - Seek to empower people by promoting awareness and manage the impact of that exposure via education materials and tools.

## Results

**Figure 1.**
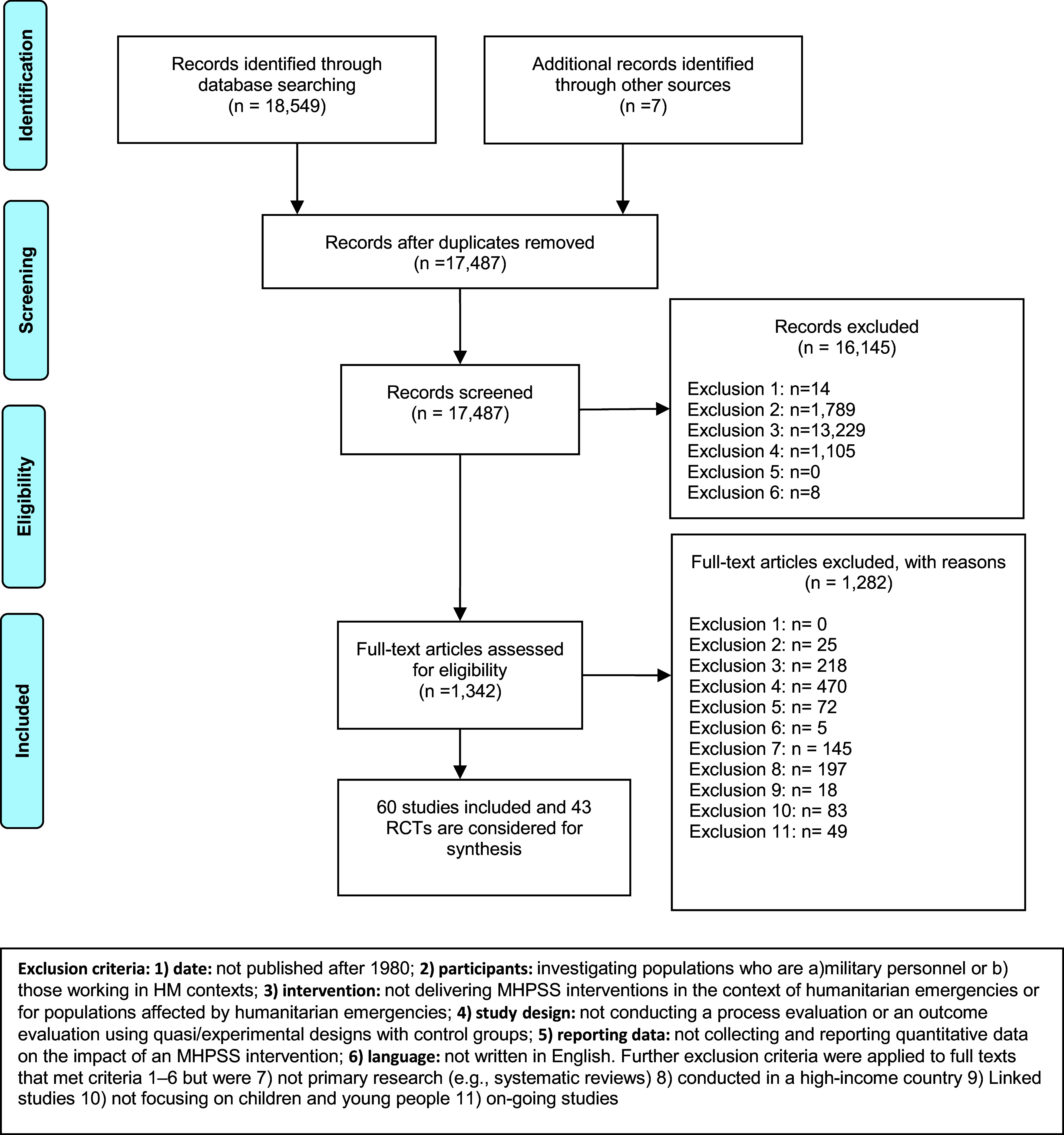
PRISMA flowchart.

We identified 18,556 records. 16,822 records were screened on title and abstract. 1,342 records were rescreened based on full-text reports. A total of 60 studies were included in the review, and 43 RCTs and cRCTs were considered for the synthesis (see Figure 1). Twelve electronic databases, key websites and citation checking were undertaken. Forty-three RCTs published in English between January 1980 and May 2023 were included in the review. Overall, the findings suggest that cognitive behavioural therapy may improve depression symptoms in children and young people affected by humanitarian emergencies (pooled ES = -0.15; 95% CI (−0.29, −0.01), I^2^ = 51.86%). Narrative exposure therapy may reduce feelings of guilt (pooled ES = −0.43, 95% CI (−0.79, −0.07), I^2^ = 0%). However, the impact of the other MHPSS modalities across outcomes is inconsistent. In some contexts, providing psychosocial programmes involving creative activities may increase the symptoms of depression in children and young people. These findings emphasise the need for the development of MHPSS programmes that can safely and effectively address the diverse needs of children and young people living in adversarial environments.

Forty-two (70%) of 60 studies were published since 2010. Most studies were conducted in the Middle East and Asia (n = 36, 60%) and 19 (33.33%) in sub-Saharan Africa. The majority of the research evidence (n = 49) was conducted in war and conflict settings. One-fifth of the studies (n = 11) evaluated the impact of MHPSS programmes on displaced and refugee children (Dybdahl, 2001; Thabet Abdel et al., 2005; Bolton et al., 2007; Ertl et al., 2011; Kalantari et al., 2012; Lange-Nielsen et al., 2012; Morris et al., 2012; Annan et al., 2017; Sirin et al., 2018; Yankey and Biswas Urmi, 2019; Fine et al., 2021). Nearly one-quarter investigated the impact of MHPSS programmes on children and young people affected by natural disasters such as earthquakes, tsunami (Goenjian et al., 2005; Schauer von, 2008; Shooshtary et al., 2008; Berger and Gelkopf, 2009; Catani et al., 2009; SHoaakazemi et al., 2012; Chen et al., 2014; Cluver, 2015; Pityaratstian et al., 2015; Akiyama and Gregorio Ernesto, 2018; Cleodora et al., 2018; Dhital et al., 2019; Nopembri et al., 2019). The majority of the studies assessed the impact of MHPSS programmes in the aftermath of disasters. Only four studies aimed to measure the impact of MHPSS programmes on young women and girls (SHoaakazemi et al., 2012; O’callaghan et al., 2013; Robjant et al., 2019; Ahmadi et al., 2023) and only one study focused on former child soldiers and war-affected boys (McMullen et al., 2013). Most MHPSS programmes were delivered in school or classroom. Additional locations included the community (Khamis and Coignez, 2004; Loughry et al., 2006; Morris et al., 2012; Betancourt et al., 2014; Annan et al., 2017; Panter-Brick et al., 2018; Sirin et al., 2018; Robjant et al., 2019; Brown et al., 2023), refugee camps (Khamis and Coignez, 2004; Thabet Abdel et al., 2005; Bolton et al., 2007; Ertl et al., 2011; Lange-Nielsen et al., 2012; Fine et al., 2021), family home (Dybdahl, 2001; Brown et al., 2009; Chen et al., 2014), outdoor areas (Richards et al., 2014; Pityaratstian et al., 2015; Akiyama and Gregorio Ernesto, 2018) or within church settings (O’callaghan et al., 2014). The majority of MHPSS programmes (n = 50, 83.33%) were conducted in group settings, while four (6.7%) were delivered in group and individual formats. Of 60 studies, 22 studies evaluated the impact of CBT; 20 studies evaluated psychosocial programmes. Other studies focused on NET (n = 10), psychoeducation (n = 3) and other interpersonal and body psychotherapy modalities (n = 11). Eight studies evaluated more than one MHPSS model.

Approximately three-quarters of the MHPSS programmes included in the review provided advice and support to children and young people by sharing dialogue and discussing experiences within groups or with specialists. Other implementation strategies included exercise, drawing, arts and crafts, relaxation and breathing techniques. Some included social activities such as sports, games, drama, film or providing life skill training. Several MHPSS programmes were designed to engage with carers and work with teachers and school management, and/or the wider community. MHPSS programme implementation varied in terms of intensity and duration. Nevertheless, MHPSS programmes designed for children and young people in low-resource, humanitarian settings were typically delivered between four to 15 sessions (n = 37), each lasting approximately 60–120 min (n = 31). Five MHPSS programmes were delivered in multiple sessions, spanning one school year or more (Layne Christopher et al., 2008; Peltonen et al., 2012; Nopembri et al., 2019; Torrente et al., 2019; Yankey and Biswas Urmi, 2019).

**Figure 2.**
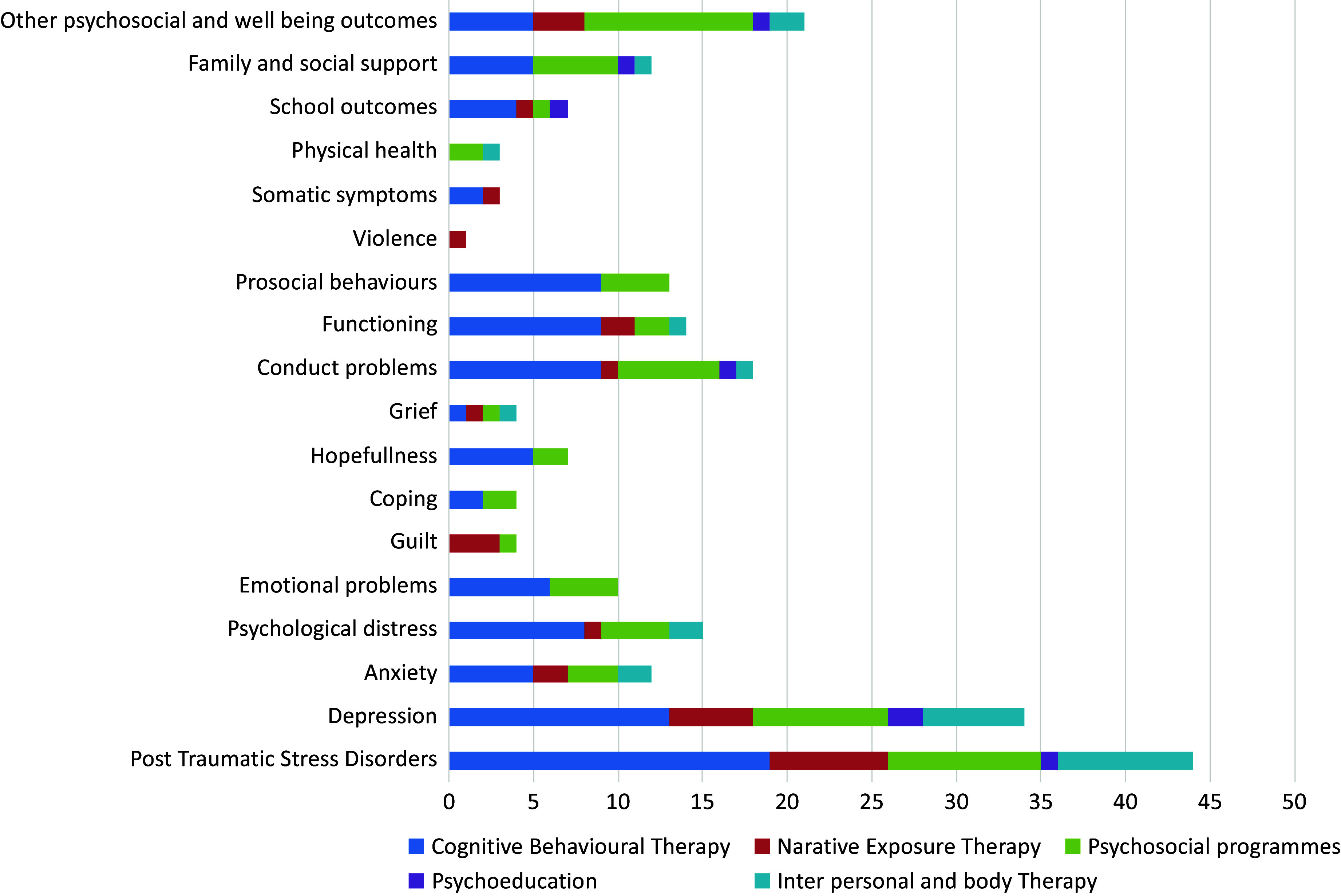
Type of MHPSS programmes and outcomes*. *More than one types of the MHPSS programmes can be evaluated in one study.

A wide range of outcomes was used to assess the impact of MHPSS programmes (see Figure 2). The most commonly reported mental health measures were PTSD (n = 41 studies, 68%) and depression (n = 31 studies, 51.67%). Other traumatic stress reactions and emotional well-being measures reported in more than ten studies included psychological distress, conduct problems, functioning, anxiety and prosocial behaviours. Other coping resources, such as family and social support outcomes, were reported in 13 studies. Less commonly reported outcome measures were emotional problems (n = 8), educational outcomes (n = 8), hopefulness (n = 7), guilt (n = 3) and grief (n = 4). We identified various tools used to measure the impact of MHPSS programmes. Nearly half of the included studies clearly explained whether and how the standardised instruments were translated into local languages or piloted for use in local settings. (the key characteristics of 60 studies are summarised in file S3).

We included 43 RCTs in the synthesis. Of the 43 RCT studies, 12 studies were clustered RCTs, assigning the participants by school (Schauer von, 2008; Tol et al., 2008; Tol et al., 2012; Tol et al., 2014; Dhital et al., 2019; Nopembri et al., 2019; Torrente et al., 2019), class (Berger and Gelkopf, 2009; Qouta Samir et al., 2012; Berger et al., 2018) or local district (Jordans et al., 2010; Fine et al., 2021). The most evaluated MHPSS programmes were CBT (n = 20) and psychosocial programmes (n = 12). We further classified the studies according to the Inter-Agency Standing Committee Guideline on MHPSS in Emergency Settings (IASC, 2007a). Two types of IASC MHPSS intervention pyramid were evaluated: one aimed to strengthen community and family support (Tier 2; n = 27), and the other focused on delivering focused, non-specialised supports to children and their families (Tier 3; n = 29). The majority of the included studies in the synthesis (n = 30, 69.76%) screened participants for mental health conditions before being enrolled in the study. Nearly all RCTs (n = 40) assessed the short-term impact (0–3 months) of MHPSS programmes with two studies assessing the impact of MHPSS programmes for more than 12 months (Schauer von, 2008; Torrente et al., 2019). Twenty-four studies compared MHPSS programmes with waitlist control groups; six with active interventions and eight with Treatment As Usual (TAU), eight studies with no intervention (Kalantari et al., 2012; SHoaakazemi et al., 2012; Betancourt et al., 2014; Chen et al., 2014; Richards et al., 2014; Berger et al., 2018; Dhital et al., 2019; Yankey and Biswas Urmi, 2019). Eight studies had more than one comparison groups (Bolton et al., 2007; Ertl et al., 2011; Chen et al., 2014; Richards et al., 2014; Cluver, 2015; O’callaghan et al., 2015; Nopembri et al., 2019; El-Khani et al., 2021). Eighteen studies were judged to be high risk of bias, 17 with medium risk of biases or having some concerns and eight with low risk of bias. (see Table 2).

**Table 2.** Characteristics of 43 RCTs

Study	Population and sample	Type of MHPSS/Control group and the ISAC intervention pyramid	Implementation	Follow ups	Outcomes reported
(Ahmadi et al., 2023) Afghanistan Armed conflict RCT ROB: High	Young girls 11–19 years old, mean age 15.96 (1.97) years N = 125	NET: Memory Training for Recovery Adolescent (METRA)/TAU Tier 3: Focused, non-specialised support	Group Private room rent property in Kabul 10 sessions, 60 min over two weeks	3 months	PTSD* Depression Anxiety Psychological distress
(Annan et al., 2017) Thailand Armed conflict RCT ROB: Some concern	Burmese migrant and displaced children Female = 51% N = 479 children and 513 caregivers	Psychosocial: Parent and Family Skills interventions adapted from the Strengthening Families Programme (SFP) originally developed in USA for substance abusing parents and their children /Waitlist Tier 2: Strengthening community and family supports	Group Community spaces such as school and community halls 12 weekly, 2-h, sessions	1, 6 months	Emotional problems Conduct problems Attention problems Child psychosocial protective factor
(Barron et al., 2016) Palestine Armed conflict and political violence RCT ROB: Low	School students; age 11–15 years (mean age = 13.57 years (0.82); Female = 59.7% N = 154	CBT: The Teaching Recovery Techniques (TRT). The group-delivered programme, based on CBT, focuses specifically on children’s symptoms of PTSD/ Waitlist Tier 3: Focused, non-specialised support	Group School 5 weekly, 90 min, session, over 5 weeks	2 Weeks	PTSD Depression Disassociation
(Berger et al., 2018) Tanzania Armed conflict cRCT ROB: High	School children, mean age: INT = 12.44 years (0.89); CON = 12.48 years (0.93); Females = 50.8% N = 183	CBT: Erase Stress Prosocial (ESPS) is a universal school-based programme divided into two sets of strategies: stress-reduction interventions and pro-social interventions (i.e., perspective-taking, empathy training, mindfulness and compassion-cultivating practices) / No intervention Tier 2: Community and family support	Group School 16 sessions, 90 min weekly	One week, 8 months	Functioning impairment Somatic symptoms Anxiety Prosocial behaviour Conduct problems Academic achievement Social relationship
(Berger and Gelkopf, 2009) Sri Lanka Tsunami Quasi-cRCT ROB: Some concerns	Elementary school students; age 9–14 years Female = 41.7% N = 166	CBT: ERASE Stress Sri Lanka (ES-SL) – a classroom-based programme designed to help children cope with the threat and the exposure to disaster and trauma. The programme involved children’s caregivers with home assignments/Waitlist Tier 2: Community and family support	Group School/classroom 12, 90 min, weekly	3 months	PTSD Depression Somatic symptoms Function impairment Hope
(Betancourt et al., 2014) Sierra Leone Armed conflict RCT ROB: Low	Youth; mean age = 18 years; Female 45.6% N = 436	CBT: The Youth Readiness Intervention combines elements drawn primarily from CBT and Interpersonal therapy, both evidence-based therapies with demonstrated effectiveness in treating depression, anxiety, and interpersonal deficits due to trauma/Waitlist Tier 2: Community and family support Tier 3: Focused, non-specialised support	Group Community sites 10–12, 90 min sessions, over 10 weeks	Post and 6 months	PTSD Emotional problems Psychological distress Functional impairment Prosocial Social support School performance and attendance
(Bolton et al., 2007) Uganda Armed conflict RCT ROB: Low	Acholi adolescent aged 14–17 years from 2 IDP camps: Female 57.3% N = 314	Group Interpersonal Psychotherapy, psychosocial: Creative play – aiming to strengthening youth’s resilience by verbal and non-verbal expression of thoughts and feelings through activities such as songs, art, role plays, music, sports, games/Waitlist Tier 3: Focused, non-specialised support	Group Camps 16, 90–120 min, sessions	One month	Depression Function impairment
(Brown et al., 2023) Lebanon Armed conflict RCT ROB: Some concerns	Children aged 10–14; Female 45% and the average age was 11.7 (SD = 1.3) N = 67 children and 67 caregivers	Early Adolescent Skills for Emotions (EASE)/TAU Tier 2: Community and family support Tier 3: Focused, non-specialised support	Group 7, weekly 90 min, sessions	Post and 3 months	PTSD Depression Psychological distress* Functioning Well-being
(Bryant et al., 2022) Jordan Armed conflict RCT ROB: Low	Young Syrian adolescents; females = 49.5%; mean age = 11.6. years (1.3) N = 471	Early Adolescent Skills for Emotions (EASE) – focus on arousal reduction, behavioural activation and problem management as these strategies have been shown to be key for reducing internalising problems in adolescents/TAU Tier 2: Community and family support Tier 3: Focused, non-specialised support	Group 7, weekly 90 min, sessions	9 weeks and 3 months	PTSD Depression Functioning Emotional problems* School outcome Conduct problems* Attention* Well being
(Catani et al., 2009) Sri Lanka Tsunami and war RCT ROB: Some concerns	Children aged 8–14 years, Female 45.16% N = 31	KIDNET – the participants constructed a detailed chronological account of his or her own biography/ Active Intervention Tier 3: Focused, non-specialised support	Individual Camps 6, 60–90 min, sessions over two weeks	4–5 weeks and 6 months	PTSD Somatic symptoms Function impairment
(Chen et al., 2014) China Earthquake RCT ROB: High	Children, Female 68% N = 40	CBT, Interpersonal and body psychotherapy / No intervention Tier 2: Community and family support Tier 3: Focused, non-specialised support	Group Home 6, 60 min, sessions, weekly	Post and 3 months	PTSD Depression Psychological resilience
(Cluver, 2015) Haiti Earthquake RCT and Non-RCT ROB: High	Children living in orphanages; mean age = 11.23 years; Female 42% N = 61	Interpersonal and body psychotherapy (Yoga) /Active intervention (Dance) Tier 3: Focused, non-specialised support	Group School 8 weeks, twice, 45 min, weekly	Post	PTSD Psychological distress
(Dawson et al., 2018) Indonesia Civil war RCT ROB: Low	Children aged between 7–14; mean age: INT = 10.50 years (0.93); CON = 10.25 years (1.16) N = 64	Trauma-focused CBT with children and caregivers/Active intervention Tier 2: Community and family support Tier 3: Focused, non-specialised support	Individual , 1 h session over 5 weeks	3 months	PTSD Depression Anger
(Dhital et al., 2019) Nepal Natural disaster cRC ROB: High	Students grade 6–8; mean age INT = 12.9 years (1.3); CON = 12.9 years (1.4) N = 1,220	Psychosocial: Teacher-mediated school-based intervention/No intervention Tier 2: Community and family support	Group School 2 days training, eight sessions, 1–2 h	6 months	PTSD Depression Grief
(Dybdahl, 2001) Bosnia and Herzegovina Armed conflict RCT ROB: High	Bosnian-displaced mothers; Female only (Mothers) – Child (mean age = 5.5 years; Female =55.17%) N = 87	Psychosocial intervention aiming to promote the development and well-being of young children through parental involvement, support and education, and parent–child relationships/ TAU Tier 2: Community and family support	Group Weekly group meeting for five months; 60 min, home visit	Post	Depression Psychological distress Well-being Cognitive performance Physical health
(El-Khani et al., 2021) Lebanon Armed conflict RCT ROB: Some concerns	Children and their caregivers: Child aged 9–12 years; N = 119 children and caregivers	CBT: Teaching Recovery Techniques (TRT) with Parenting skills (TRT + P)/TRT/Waitlist Tier 2: Community and family support Tier 3: Focused, non-specialised support	Group Five weekly sessions; 120 min for each session 2 parent sessions	2 weeks and 12 weeks	Depression Anxiety Conduct behaviour
(Ertl et al., 2011) Uganda Civil war RCT ROB: Some concerns	Child soldiers; Female 67.1% N = 1,113	Narrative Exposure Therapy (NET) -a short term, trauma-focused treatment, Psychosocial/ Waitlist Tier 2: Community and family support Tier 3: Focused, non-specialised support	Individual Camp 8 sessions, 90–120 min, 3 times a week	3, 6, 12 months	PTSD* Depression Guilt Suicide risks Function impairment Stigmatisation
(Fine et al., 2021) Tanzania Armed conflict cRCT ROB: Some concerns	Young adolescents in refugee camp Female 49%; mean age = 12.3(1.5) N = 82 children, N = 64 caregivers	The Early Adolescent Skills for Emotions (EASE) intervention aiming to reduce symptoms of internalising disorders, including depression and anxiety/TAU Tier 2: Community and family support Tier 3: Focused, non-specialised support	7 weekly, 90 min, sessions	1 week	PTSD Psychological Distress* Functioning Prosocial Somatic symptoms Well being
(Getanda and Vostanis, 2020) Kenya Displacement RCT ROB: Some concerns	Youth aged 14–17 years; N = 54	NET: Writing for Recovery – focusing on sensory aspects of traumatic events leading to PTSD and other symptoms of distress/Waitlist Tier 2: Community and family support Tier 3: Focused, non-specialised support	6 sessions over 3 days	Post	PTSD Depression Anxiety Quality of life
(Gordon et al., 2008) Kosovo Armed conflict RCT ROB: High	Children; mean age = 16.3 years; Female = 75.60% N = 82	Interpersonal and body psychotherapy: Mind–body technique model – a combination of a number of mind–body modalities with self-expression (spoken, written words, drawings, movement)/ Waitlist Tier 2: Community and family support Tier 3: Focused, non-specialised support	Group School 12, 120 min, session, twice weekly for 6 weeks	Post	PTSD
(Jordans et al., 2010) Nepal Armed conflict cRCT ROB: Some concerns	School children; mean age = 12.7 years; girls 48.6% N = 325	CBT: A school-based psychosocial intervention – the Classroom-Based Intervention (CBI) – an eclectic intervention based on concepts from creative-expressive and experimental therapy, cooperative play and CBT/ Waitlist Tier 2: Community and family support Tier 3: Focused, non-specialised support	Group Classroom 15, 60 min, sessions over 5 weeks	Post	PTSD* Depression* Anxiety* Psychological distress* Conduct problems* Functional impairment* Prosocial Hope
(Kalantari et al., 2012) Iran Armed conflict RCT ROB: High	Afghan refugees in school; mean age INT = 14.58 years; CON = 15.03 years; Females 55% N = 64	NET: Writing For Recovery – the writing sessions developed for adolescents who have experienced a trauma/ No intervention Tier 3: Focused, non-specialised support	Group School 3 consecutive days: two, 15 min, sessions, a 1 day	Post	**Grief**
(Khamis and Coignez, 2004) Palestine Armed conflict RCT ROB: High	Children aged 6–16 years, Female 43.37% N = 664	CBT: Classroom-based intervention-a psychosocial integration and recovery programme for children and adolescents and their adult caregivers who are exposed to psychological trauma/ Waitlist Tier 2: Community and family support	Group School/camp 15 sessions over 5 weeks	Post	Emotional problems Anxiety Conduct problems Prosocial Coping Hope Family relationship Peer and sibling relationship School performance Self-esteem
(Lange-Nielsen et al., 2012) Gaza Armed conflict RCT ROB: High	Adolescents; 12–17 years mean age = 14.54 years (1.47); Female 50% N = 124	NET: Writing For Recovery-a manual-based group intervention aimed at adolescents who have a history of trauma through short writing sessions/Waitlist Tier 3: Focused, non-specialised support	Group School 2, 15 min, sessions per day for 3 days	Post, one mo,5 months	PTSD Depression Anxiety
(Layne Christopher et al., 2008) Indonesia Terrorist attack RCT ROB: High	Children (Female 52.7%) mean age = 9.83 years (1.53) N = 127	Interpersonal and body psychotherapy: A spiritual-hypnosis assisted therapy/ Active intervention Tier 3: Focused, non-specialised support	Group School 17–20 weekly group sessions for 7 months (school year), between 60 to 90 min	Post and 4 months	PTSD Depression Grief
(McMullen et al., 2013) Congo Armed conflict RCT ROB: Some concerns	39 former soldiers and 11 war-affected boys; mean age = 15.8 years N = 50	A manualised, Trauma-focused CBT/Waitlist Tier 3: Focused, non-specialised support	Group School 15 sessions	7 weeks and 3 months	PTSD Emotional problems Psychological distress Conduct problems Prosocial
(Nopembri et al., 2019) Indonesia Natural disaster cRCT ROB: High	Students: INT = mean age 10.4–10.39 years N = 810	Psychosocial: Physical Education and sports classes/TAU Tier 2: Community and family support	Group School 15, 2 h sessions, 3 days a week over five weeks	Post	Coping Problem solving skills
(O’callaghan et al., 2013) Congo Armed conflict RCT ROB: Low	Girls who had witnessed or had personal experience of rape of sexual abuse; mean age = 16 years N = 52	A manualised, culturally modified, trauma focused CBT/Waitlist Tier 3: Focused, non-specialised support	Group School 15, 120 min, sessions, 3 days a week over five weeks	7 weeks and 3 months	PTSD* Emotional problems Conduct problems Prosocial
(O’callaghan et al., 2014) Uganda Armed conflict RCT ROB: Some concerns	Children: mean age = 13.42 years; females 45% N = 159	A manualised, family-focused – psychosocial interventions/ Waitlist Tier 2: Community and family support	Group Church 3 times weekly, 8, 12o mins, sessions over 4 weeks	Post and 3 months	PTSD * Emotional problems* Conduct problems* Prosocial*
(O’callaghan et al., 2015) Congo Armed conflict RCT ROB: Low	War affected youth; mean age = 14.88 years N = 50	Trauma-focused CBT – a combination of cognitive therapy and behavioural therapy Psychosocial: Child Friendly Spaces – a psychosocial intervention aiming to improve resilience and well-being of youth through community-based, structured activities held in a safe, child friendly environment/ Active intervention Tier 2: Community and family support Tier 3: Focused, non-specialised support	Group Field attached to local schools 9, 90 min, sessions, three sessions per week plus two, 90 min caregivers’ sessions in group sessions	Post and 6 months	PTSD Emotional problems Conduct problems Prosocial
(Panter-Brick et al., 2018) Syria Conflict RCT ROB = Low risk	Adolescents, mean age = 14.37 years (1.72), Female = 43% N = 603	Psychosocial: Advancing adolescents programme – consisting of structural activities that aimed to promote capacities for the mediation of extreme and prolonged stress/Waitlist Tier 2: Community and family support	Group community based (Youth centres, designed as ‘Adolescent Friendly Spaces) 16 sessions (twice per week) for 8 weeks	10 weeks, 11 months	PTSD Emotional problems Human distress* Stress* Prosocial Human insecurity*
(Pityaratstian et al., 2015) Thailand Tsunami RCT ROB: Some concerns	Children; mean age = 12.25 years: Female = 72.2% N = 36	CBT – a manual-based and adapted from the Teaching Recovery Techniques (TRT)/Waitlist Tier 3: Focused, non-specialised support	Group School and outdoor Daily, 120 min, sessions for 3 days	Post and 1 month	PTSD
(Qouta Samir et al., 2012) Palestine Armed conflict cRCT ROB: Some concerns	Children; mean age = 11.29 years; Female 49.4% N = 482	CBT: Extra curriculum sessions of the Teaching recovering techniques (TRT) / Waitlist Tier 2: Community and family support	Group School 2, weekly, 2 h session in total of 16 sessions, last for 4 weeks	Post and 6 months	PTSD Depression Psychological distress Prosocial Family factor Peer and sibling relation Well-being Maternal attachment Family atmosphere
(Richards et al., 2014) Uganda Armed conflict RCT and non-RCT ROB: Some concerns	Children; mean age = 9.83 years N = 1,462 Intervention boys =74, girls 81; waitlist boys = 72	Psychosocial: voluntary competitive sports for development football league/Waitlist and no intervention Tier 2: Community and family support	Group Sport field 11 weeks – 45 min per session	Post	Depression Anxiety Physical health
(Robjant et al., 2019) DRC Armed conflict RCT ROB: Some concerns	92 female former child soldiers, age range 11–25 years N = 92	Narrative Exposure Therapy adapted for offenders (FORNET)/TAU Tier 3: Focused, non-specialised support	Group and individual Community 6 individual sessions, 90–120 min; group therapy weekly for 60–90 min	3, 9 months	PTSD* Depression Conduct problem Violence behaviour Guilt Social acknowledgement
(Schauer von, 2008) Sri Lanka Armed conflict and Tsunami cRCT ROB: Some concerns	Children who suffered severe PTSD; mean age = 13.1 years N = 47	Manualised KIDNET – a trauma-focused, short-term therapy with exposure elements/TAU Tier 3: Focused, non-specialised support	Group School 6, 60–90 min, sessions	5 months post, and 13 months post-int.	PTSD School performance
(SHoaakazemi et al., 2012) Iran Earthquake RCT ROB: High	Girls with PTSD (15–18 years) N = 24	Interpersonal and body psychotherapy: Logo therapy/ No intervention Tier 3: Focused, non-specialised support	Individual 8,60 min, sessions	Post	Quality of life
(Sirin et al., 2018) Turkey Conflict RCT ROB: High	Children from five refugee camps N = 147	Psychoeducation: Project Hope: an online-game based learning intervention/ Waitlist Tier 2: Community and family support	Group Community 2 h, 5 days a week for 4 weeks	Post	Common mental health disorder School performance Cognitive skills
(Tol et al., 2008) Indonesia Political violence cRCT ROB: Some concerns	School children, mean age = 9.9 y, Girls 48.63% N = 403	CBT: School-based mental health including trauma-processing activities, cooperative play, and creative expressive elements/Waitlist Tier 3: Focused, non-specialised support	Group Classroom 15 sessions, over 5 weeks	Post and 6 months	PTSD Depression Anxiety Conduct problems Functional impairment Hope
(Tol et al., 2012) Sri Lanka Armed conflict cRCT ROB: Some concerns	Children; mean age 12.29 years; Girls 48% N = 399	CBT: School-based mental health – consisting of CBT and creative expressive elements/Waitlist Tier 3: Focused, non-specialised support	Group School 15 sessions, over 5 weeks	One week and 3 months	PTSD* Depression* Anxiety* Psychological distress Conduct problems Function impairment Prosocial Coping
(Tol et al., 2014) Burundi Armed conflict cRCT ROB: High	Children, mean age 12.29 years N = 329	CBT: The classroom-based intervention (CBI) including universal preventive activities and provision of mental health treatments/Waitlist Tier 2: Community and family support Tier 3: Focused, non-specialised support	Group School 15 sessions, over 5 weeks	Post and 3 months	PTSD* Depression* Anxiety* Function* impairment Social support Coping Hope
(Torrente et al., 2019) DRC Armed conflict cRCT ROB: High	76 clusters and 126 schools 47.5% females, mean age = 11.66 years (1.9) N = 8,813	Psychosocial: Learning in a Healing Classroom – a universal school-based intervention to support children’s social–emotional well-being and academic learning/ Waitlist Tier 2: Community and family support	Group School One to two academic years	One year	School performance Well-being Teacher and school-level outcomes
(Yankey and Biswas Urmi, 2019) India Political violence, refugees RCT ROB: High	Tibetan refugees, age 13–17 years N = 300	Psychosocial: school-based life skill training programme/No intervention Tier 2: Community and family support	Group School 30 sessions over 11 months	2 weeks post-intervention	Coping Self-confidence Emotional intelligence

Abbreviations: CBT, cognitive behavioural therapy; cRCT, cluster randomised controlled trial; DRC, democratic republic of the congo; N, sample size; NET, narrative exposure therapy; PTSD, post-traumatic stress disorder; RCT, randomised controlled trial; ROB, risk of bias; TAU, treatment as usual; *, primary outcome when reported.

Due to a substantive amount of heterogeneity, we were able to perform a meta-analysis and found a significant positive impact of MHPSS on grief (2 studies; pooled ES = −0.55*, 95% CI (−0.91, −0.19), I^2^ = 0%), and guilt (2 studies; pooled ES = −0.51*, 95% CI (−0.83, −0.19), I^2^ = 0%). In other outcomes, we reported a range of effect sizes, presenting mixed results across studies (see S4).

**Figure 3.**
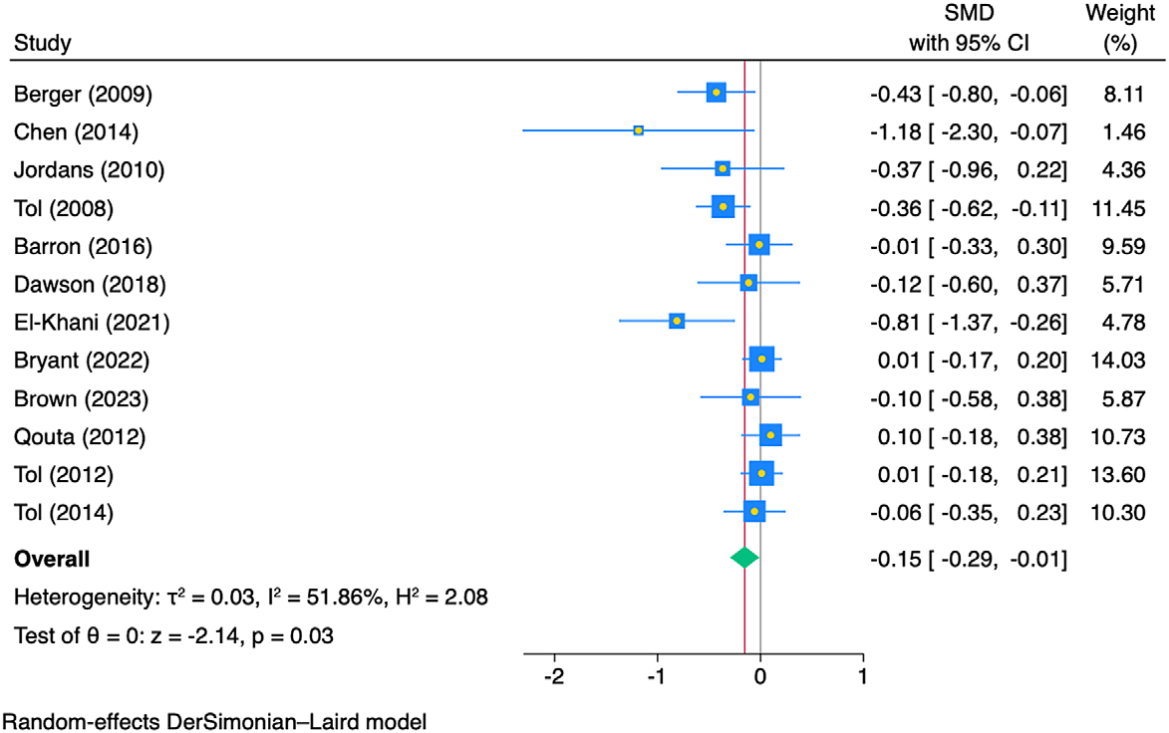
Impact of CBT programmes on depression in children and young people (n = 12 studies).

We performed an explorative analysis to assess the impact of MHPSS programmes by programme type. CBTs were evaluated in 20 studies (six low risk of bias, ten some concerns, four high risk of bias). All but two CBT programmes were delivered in a group format (Chen et al., 2014; Dawson et al., 2018). The majority of the studies evaluated the impact of CBT delivered to children in conflict-affected settings, with three studies assessed its effects on children affected by earthquakes and tsunamis (Berger and Gelkopf, 2009; Chen et al., 2014; Pityaratstian et al., 2015). These CBT programmes were primarily delivered in school settings, with two conducted in refugee camps (Khamis and Coignez, 2004; Fine et al., 2021), and one delivered at home(Chen et al., 2014). In three studies, the effect of culturally adapted, school-based trauma-based CBT designed for war-affected youth in Democratic Republic of the Congo (DRC) was examined, showing a significant reduction in PTSD, conduct and emotional problems (McMullen et al., 2013; O’callaghan et al., 2013; O’callaghan et al., 2015). The finding from the meta-analysis suggested that CBT programmes have a potential to improve depression symptoms (pooled SMD = −0.15; 95% CI (−0.29, −0.01, I^2^ = 51.86%%) (Figure 3). We did not perform a meta-analysis of the effects of CBT on other outcomes due to heterogeneity.

We identified 11 RCTs (two low risk of bias, four some concerns, five high risk of bias) assessing the impact of psychosocial interventions (Dybdahl, 2001; Bolton et al., 2007; Ertl et al., 2011; O’callaghan et al., 2014; Richards et al., 2014; O’callaghan et al., 2015; Annan et al., 2017; Panter-Brick et al., 2018; Dhital et al., 2019; Nopembri et al., 2019; Torrente et al., 2019; Yankey and Biswas Urmi, 2019). These psychosocial programmes were varied in their programme design. Three of the interventions were delivered in Uganda, evaluating a creative play programme (Bolton et al., 2007), competitive sport and games (Richards et al., 2014) and academic writing (Ertl et al., 2011). Two psychosocial programmes were implemented in DRC involving community, school and family (O’callaghan et al., 2014; O’callaghan et al., 2015; Torrente et al., 2019). These programmes used life skill training to address discrimination and to develop coping skills (O’callaghan et al., 2014; Panter-Brick et al., 2018; Nopembri et al., 2019; Yankey and Biswas Urmi, 2019). One study evaluated the effect of psychosocial support for mothers in conflict-affected Bosnia and Herzegovina to improve mental health outcomes of their children (Dybdahl, 2001). Overall, the evidence is inconclusive regarding the impact of psychosocial programmes on children and young people’s mental health outcomes. There is no statistically significant improvement from participating psychosocial programmes on PTSD (n = 3 studies; pooled SMD = −0.18, CI 95% (−0.44,0.08) I^2^ = 39.07%), conduct problems (n = 2 studies, pooled SMD = 0.02, 95% CI (−0.18, 0.22), I^2^ = 30.03%), emotional problems (n = 2 studies; pooled SMD = −0.00, 95% CI (−0.16,0.15), I^2^ = 0%) or functioning (n = 2 studies, pooled SMD = 0.03, 95% CI (−0.29, 0.34), I^2^ = 0%). However, it is important to point out that the findings from the meta-analysis showed unintended impact of psychosocial programmes on depression symptoms (n = 5 studies pooled SMD = 0.17, 95% CI (0.00, 0.35), I^2^ = 14.49%). Three of five studies evaluated psychosocial programmes, aiming to engage children and young people with different activities such as songs, arts, sports or academic catch-up (Bolton et al., 2007; Ertl et al., 2011; Richards et al., 2014). The other psychosocial programmes delivered the programmes to parents and teachers to support interactions with children and young people who affected by war (Dybdahl, 2001) and earthquake (Dhital et al., 2019).

Eight studies (four some concerns, four high risk of bias) evaluating the impact of narrative exposure therapy (NET) were included in the review.(Schauer von, 2008; Catani et al., 2009; Ertl et al., 2011; Kalantari et al., 2012; Lange-Nielsen et al., 2012; Robjant et al., 2019; Getanda and Vostanis, 2020; Ahmadi et al., 2023). Six NET programmes were delivered in a group format (Schauer von, 2008, Kalantari et al., 2012, Lange-Nielsen et al., 2012, Robjant et al., 2019, Getanda and Vostanis, 2020, Ahmadi et al., 2023), with three delivered to individual participants.(Catani et al., 2009; Ertl et al., 2011; Robjant et al., 2019). Two studies were carried out in Sri Lanka: one with children affected by civil war (Schauer von, 2008) and the other one was carried out immediately after the 2004 tsunami (Catani et al., 2009). Two were carried out in camps for internally displaced persons (IDPs): one in Palestine (Lange-Nielsen et al., 2012) and the other in Uganda (Ertl et al., 2011). One study was conducted in a refugee camp in Iran (Kalantari et al., 2012). One study evaluated the NET programme delivered to female former child soldiers in DRC (Robjant et al., 2019). Another study evaluated the Memory Training for Recovery-Adolescent Intervention delivered to Afghan adolescent girls. The findings from the meta-analysis indicate that NET may have a significant impact in reducing the feelings of guilt (n = 2 studies; pooled SMD = -0.43, 95% CI (−0.79, − 0.07), I^2^ = 0%). However, NET may have no statistically significant impact on other internalised symptoms or functioning. One randomised study assessing the impact of the Writing for Recovery (WfR) programme in Gaza found that the children in the intervention group experienced an increase in depression and anxiety symptoms compared to the wait-list control group (Lange-Nielsen et al., 2012).

In addition, we identified six studies (one low risk of bias, five high risk of bias) evaluating six interpersonal and body psychotherapy programmes including Group Interpersonal Psychotherapy (IPT-G) (Bolton et al., 2007), counselling (Chen et al., 2014), yoga (Cluver, 2015), mind and body technique (Gordon et al., 2008), logotherapy (SHoaakazemi et al., 2012) and school-based psychotherapy (Layne Christopher et al., 2008). The studies were carried out in five different countries (Bosnia and Herzegovina, China, Haiti, Uganda and Kosovo) affected by armed conflict (Bolton et al., 2007; Gordon et al., 2008; Layne Christopher et al., 2008) or natural disaster (SHoaakazemi et al., 2012; Chen et al., 2014). The most common outcomes reported in this group of programmes were PTSD and depression. We did not carry out statistical syntheses on these outcome measures because of differences and variations in psychotherapeutic programme modalities and intervention approaches. Four studies reported unadjusted mean scores and standard deviations of PTSD (Gordon et al., 2008; Layne Christopher et al., 2008; Chen et al., 2014; Cluver, 2015). One study evaluating a mind–body skills group in Kosovo found a significant impact of the intervention on PTSD (Gordon et al., 2008). The other studies suggested mixed findings for the interventions. Chen et al. (2014) found that support group counselling may have had little impact on PTSD in children and young people affected by the earthquake in China compared with those who received no intervention. The findings from the Layne Christopher et al. (2008) study also suggested that there might be little impact from a school-based psychotherapy intervention on schoolchildren in Bosnia. However, Cluver (2015) found that yoga may increase PTSD in children and young people compared with those in an aerobic dance group.

Three studies assessed the impact of other psychotherapy interventions on depression (Bolton et al., 2007; Layne Christopher et al., 2008; Chen et al., 2014). Only Bolton et al.’s (2007) study evaluating an IPT-G programme reported a significant positive impact of the intervention on depression. In this study, 314 Acholi children aged 14–17 from two internally displaced person camps in Northern Uganda were randomly assigned to IPT-G, creative play, or a wait-list control group. At post-intervention, the IPT-G participants showed a greater reduction in depression symptoms than those in the wait-list control group.

## Conclusions and discussion

In the last decade, considerable attempts have been made to support children and young people’s mental and psychosocial health in humanitarian emergencies (Barbui et al., 2020). Delivering CBT programmes has been suggested as a suitable choice of MHPSS programmes to reduce the symptoms of PTSD in adults across various humanitarian contexts (Bangpan et al., 2019). However, the findings from this systematic review suggest inconclusive evidence regarding the effectiveness of other MHPSS modalities, such as NET or other psychotherapies, in improving internalising symptoms in children and young people. Similar results found in recent systematic reviews evaluating psychological therapies and social interventions suggest limited evidence of the programme impact on mental health of children and young people affected by humanitarian settings (Purgato et al., 2018a; Papola et al., 2020). In addition, we found that studies evaluating psychosocial programmes incorporating components such as social support, child-friendly spaces, creative play, sports, games or academic catch ups reported potential unintended consequences. Future research should employ rigorous evaluation designs considering contextual and structural influences to better understand how these types of programming can be safely and effectively delivered to this specific population group in humanitarian settings.

We recognise that MHPSS programmes implemented in humanitarian settings may face several implementation challenges, and the observed unintended impact could arise from the interplay between programme delivery and socially and culturally sensitive contexts (Koch and Schulpen, 2018). Moreover, challenges in recruiting, retraining and training programme personnel in low-resource settings, coupled with ongoing risks and insecurity, may affect the fidelity of programme implementation (Dickson and Bangpan, 2018). Recent research highlights the possibilities of training lay community workers to deliver MHPPS programme in resource-limited settings. Future research should investigate the effectiveness of this task shifting approach to guide the advancement of the implementation of MHPSS programmes (Cohen and Yaeger, 2021). In addition, some point out that children and young people face daily threats and ongoing stressful events in humanitarian emergencies which may require access to a wide range of basic social services. Therefore, further consideration to the importance of socio-ecological and multi-sectoral programming, as part of MHPSS programme design and delivery, aiming to prevent, treat and promote mental health of populations affected by humanitarian crises, is warranted (Purgato et al., 2018a; Kamali et al., 2020; Papola et al., 2020; Tol et al., 2020; Raslan et al., 2021; Papola et al., 2022). Future research might also benefit from considering the social determinants of mental health and developing a theory of change to understand mechanisms that improve mental health outcomes and well-being throughout the life course (Allen et al., 2014).

The current systematic review offers an overview of the current state of evidence on the impact of MHPSS programmes on children and young people affected by humanitarian emergencies in LMICs. We assessed the impact of different types and modalities of MHPSS programmes, offering insights into their potential benefits and unintended consequences. Although our comprehensive search identified substantial evidence in the field, we noted some caveats when interpreting the findings. First, the quality of the studies included in the systematic review varied, with the majority judged to have some quality concerns or being at a high risk of bias. This limitation of the existing evidence is also expressed by recent systematic reviews highlighting a low quality of evidence on the impact of the programmes aiming to prevent and treat mental health in children and young people affected by humanitarian settings (Purgato et al., 2018a; Papola et al., 2020). Second, we included only studies published in English. Other high-quality studies published in other languages may provide further evidence on the impact of MHPSS programmes, especially studies from Central and South America. Third, we identified a wide range of outcomes using various scales, reflecting the attempt to adapt tools for measuring the mental health and wellbeing outcomes across socio-cultural settings. Finally, the studies included in the meta-analysis were heterogeneous, drawing on a broad evidence base that aims to evaluate the impact of multi-component, multi-level MHPSS programmes on various outcomes across humanitarian settings. Future development and evaluation of MHPSS programmes would benefit from engaging with key stakeholders and local communities to understand and theorise how interventions intend to work and how socio-cultural factors might influence their observed impact (Kneale et al., 2020; Miller et al., 2021).

We systematically reviewed research evaluating the impact of MHPSS programmes on children and young people affected by humanitarian emergencies in LMICs. Sixty studies met the inclusion criteria. We identified several research gaps. In sub-Saharan Africa, although more than one-fifth of global refugees and internally displaced persons are hosted in countries such as the Central African Republic (CAR), the Democratic Republic of the Congo (DRC), Somalia and South Sudan (UNHCR, 2018), our systematic review identifies the paucity of rigorous evidence to inform the design and implementation of MHPSS programmes in this region, in line with the recent meta-review focusing on vulnerable African children (Katsonga-Phiri et al., 2019). Secondly, there is increasing recognition of the need to develop MHPSS programmes that are tailored to girls’ and boys’ needs, considering the social determinants of their individual psychological health and well-being (Purgato et al., 2018b; Raslan et al., 2021; Lasater et al., 2022). Yet, we identified limited evidence examining the impact of gender-specific MHPSS programmes. Furthermore, we did not come across any studies explicitly targeting children with disabilities. There is a need to further develop inclusive MHPSS programmes that not only address the unique needs of children with disabilities but also consider the influence of intersectionality and factors, such as gender, culture and religion. Additional research gap identified from this systematic review includes evaluations of MHPSS programmes aiming to protect children and young people’s well-being in humanitarian settings through basic service and security provision. Finally, future evaluative research should aim to capture the long-term impact and assess the cost-effectiveness of MHPSS programmes (Purgato et al., 2018a; Papola et al., 2022).

## Supporting information

Bangpan et al. supplementary material 1 Bangpan et al. supplementary material

Bangpan et al. supplementary material 2 Bangpan et al. supplementary material

Bangpan et al. supplementary material 3 Bangpan et al. supplementary material

Bangpan et al. supplementary material 4 Bangpan et al. supplementary material

Bangpan et al. supplementary material 5 Bangpan et al. supplementary material

Bangpan et al. supplementary material 6 Bangpan et al. supplementary material

## Data Availability

The authors confirm that the data supporting the findings of this study are available within the article, references and/or its Supplementary Materials.

## Footnotes

1
https://datahelpdesk.worldbank.org/knowledgebase/articles/906519-world-bank-country-and-lending-groups.

## Supplementary material

The supplementary material for this article can be found at http://doi.org/10.1017/gmh.2024.17.
