# Supplementary material for: The impact of mental health and psychosocial support programmes on children and young people’s mental health in the context of humanitarian emergencies in low- and middle-income countries: A systematic review and meta-analysis
Source: Glob Ment Health (Camb). 2024 Feb 12;11:e21. doi: 10.1017/gmh.2024.17 (PMC10988149; doi:10.1017/gmh.2024.17)
Supplement: Bangpan et al. supplementary material 1 — Bangpan et al. supplementary material [file S2054425124000177sup001.docx]

Examples of search strategy

**Medline search**

| 1. exp Clinical Trials as Topic/ or controlled trial*.mp. or exp Randomized Controlled Trials as Topic/ |  |
| --- | --- |
| 2. Interrupted time series analysis.mp. or exp Interrupted Time Series Analysis/ |  |
| 3. (Controlled before and after stud*).mp. [mp=title, abstract, original title, name of substance word, subject heading word, keyword heading word, protocol supplementary concept word, rare disease supplementary concept word, unique identifier] |  |
| 4. Pragmatic clinical trial*.mp. |  |
| 5. program evaluation.mp. or exp Program Evaluation/ |  |
| 6. exp Pilot Projects/ or pilot scheme*.mp. |  |
| 7. Outcome evaluation.mp. or exp Evaluation Studies as Topic/ |  |
| 8. Pilot stud*.mp. |  |
| 9. exp Feasibility Studies/ or Feasibility stud*.mp. |  |
| 10. Effectiveness intervention*.mp. |  |
| 11. exp "Outcome and Process Assessment (Health Care)"/ or exp "Outcome Assessment (Health Care)"/ or Outcome assessment.mp. |  |
| 12. Process assessment.mp. |  |
| 13. Control group*.mp. |  |
| 14. comparison group*.mp. |  |
| 15. Comparison stud*.mp. |  |
| 16. Repeated measure*.mp. |  |
| 17. Performance assessment.mp. |  |
| 18. Cross over trial*.mp. |  |
| 19. exp Double-Blind Method/ |  |
| 20. Quasi experiment*.mp. |  |
| 21. policy experiment*.mp. |  |
| 22. Natural experiment*.mp. |  |
| 23. Social experiment*.mp. |  |
| 24. 1 or 2 or 3 or 4 or 5 or 6 or 7 or 8 or 9 or 10 or 11 or 12 or 13 or 14 or 15 or 16 or 17 or 18 or 19 or 20 or 21 or 22 or 23 |  |
| 25. armed conflict*.mp. |  |
| 26. post conflict*.mp. |  |
| 27. conflict affected.mp. |  |
| 28. mass conflict*.mp. |  |
| 29. War/ or exp War Crimes/ or war.mp. |  |
| 30. conflict-related.mp. |  |
| 31. civil war.mp. |  |
| 32. ('war-exposed' or 'war-affected').mp. [mp=title, abstract, original title, name of substance word, subject heading word, keyword heading word, protocol supplementary concept word, rare disease supplementary concept word, unique identifier] |  |
| 33. postwar.mp. |  |
| 34. postconflict*.mp. |  |
| 35. Displacement.mp. or exp "Displacement (Psychology)"/ |  |
| 36. exp Refugees/ or refugee*.mp. |  |
| 37. Mass killing.mp. |  |
| 38. Genocide.mp. or exp Genocide/ |  |
| 39. Disaster*.mp. or exp Disasters/ |  |
| 40. Natural disaster*.mp. |  |
| 41. Earthquakes/ or Earthquake*.mp. |  |
| 42. Typhoon*.mp. |  |
| 43. exp Droughts/ or Drought*.mp. |  |
| 44. exp Floods/ or Flood*.mp. |  |
| 45. Industrial disaster*.mp. |  |
| 46. Political violence.mp. |  |
| 47. exp Relief Work/ or Humanitarian.mp. |  |
| 48. Hurricane.mp. or exp Cyclonic Storms/ |  |
| 49. displaced population*.mp. |  |
| 50. displaced person.mp. |  |
| 51. mass adversity.mp. |  |
| 52. Industrial accident*.mp. |  |
| 53. exp Volcanic Eruptions/ or Volcano*.mp. |  |
| 54. Landslide*/ or landslide*.mp. |  |
| 55. Avalanche*.mp. or exp Avalanches/ |  |
| 56. exp Tsunamis/ or Tsunami*.mp. |  |
| 57. Storm surge*.mp. |  |
| 58. Tornado*.mp. |  |
| 59. Cyclone*.mp. |  |
| 60. Infestation*.mp. |  |
| 61. Wildfire.mp. |  |
| 62. extreme temperature.mp. |  |
| 63. exp Terrorism/ or Terrorist attack*.mp. or exp Bioterrorism/ |  |
| 64. 25 or 26 or 27 or 28 or 29 or 30 or 31 or 32 or 33 or 34 or 35 or 36 or 37 or 38 or 39 or 40 or 41 or 42 or 43 or 44 or 45 or 46 or 47 or 48 or 49 or 50 or 51 or 52 or 53 or 54 or 55 or 56 or 57 or 58 or 59 or 60 or 61 or 62 or 63 |  |
| 65. Mental health*.mp. or exp Mental Health/ |  |
| 66. exp Mental Disorders/ or psychosocial.mp. or exp Depressive Disorder/ or exp Psychosocial Deprivation/ or exp Adaptation, Psychological/ or exp Social Adjustment/ or exp Stress, Psychological/ |  |
| 67. exp Psychiatric Somatic Therapies/ or psychiatric.mp. or exp Social Work, Psychiatric/ |  |
| 68. exp Psychotherapy, Rational-Emotive/ or exp Psychotherapy/ or exp Psychotherapy, Multiple/ or exp Psychotherapy, Group/ or psychotherapy.mp. or exp Psychotherapy, Brief/ or exp Psychotherapy, Psychodynamic/ |  |
| 69. exp Depression/ or exp Cognitive Therapy/ or exp Stress Disorders, Post-Traumatic/ or Psychological treatment*.mp. or exp Behavior Therapy/ |  |
| 70. Mental health service*.mp. or exp Mental Health Services/ |  |
| 71. Social support.mp. or exp Social Support/ |  |
| 72. exp Anxiety Disorders/ or Cognitive Behavioural Therap*.mp. |  |
| 73. Community-based psychosocial support.mp. |  |
| 74. exp Counseling/ or Counselling.mp. |  |
| 75. counseling.mp. or Counseling/ |  |
| 76. Cognitive processing therap*.mp. |  |
| 77. exp Art Therapy/ or Creative arts.mp. |  |
| 78. Debriefing.mp. or exp Crisis Intervention/ |  |
| 79. Economic support.mp. |  |
| 80. Exposure therap*.mp. or exp Implosive Therapy/ |  |
| 81. (Eye movement Desensitization and Reprocessing).mp. [mp=title, abstract, original title, name of substance word, subject heading word, keyword heading word, protocol supplementary concept word, rare disease supplementary concept word, unique identifier] |  |
| 82. Eclectic.mp. |  |
| 83. Group therap*.mp. |  |
| 84. Interpersonal therap*.mp. |  |
| 85. Grief Intervention*.mp. |  |
| 86. Family therap*.mp. or Family Therapy/ |  |
| 87. family-based intervention*.mp. |  |
| 88. Narrative exposure therap*.mp. |  |
| 89. Music therap*.mp. or exp Music Therapy/ |  |
| 90. Psychological intervention*.mp. |  |
| 91. Psychosocial care intervention*.mp. |  |
| 92. exp Relaxation/ or Relaxation.mp. or exp Relaxation Therapy/ |  |
| 93. Preventive psychosocial intervention*.mp. |  |
| 94. Psychodynamic therap*.mp. |  |
| 95. Skill based group*.mp. or exp Health Education/ |  |
| 96. Safe space.mp. |  |
| 97. psychoeducation.mp. |  |
| 98. Trauma focused intervention*.mp. |  |
| 99. Thought field therap*.mp. |  |
| 100. (Dance and movement therap*).mp. [mp=title, abstract, original title, name of substance word, subject heading word, keyword heading word, protocol supplementary concept word, rare disease supplementary concept word, unique identifier] |  |
| 101. Prolonged exposure therap*.mp. |  |
| 102. School-based.mp. |  |
| 103. Stress Inoculation Therap*.mp. |  |
| 104. KIDNET.mp. |  |
| 105. exp Psychophysiologic Disorders/ or Specialised psychotherapeutic intervention.mp. |  |
| 106. Interpersonal psychotherapy.mp. |  |
| 107. Testimony Therap*.mp. |  |
| 108. Trauma healing.mp. |  |
| 109. Reconciliation.mp. |  |
| 110. Psychopharmacological treatment*.mp. |  |
| 111. Physiotherapy.mp. |  |
| 112. Psychological care.mp. |  |
| 113. exp Home Care Services/ or exp Self Care/ |  |
| 114. (Sport and recreation).mp. [mp=title, abstract, original title, name of substance word, subject heading word, keyword heading word, protocol supplementary concept word, rare disease supplementary concept word, unique identifier] |  |
| 115. Case management.mp. or exp Case Management/ or exp "Referral and Consultation"/ |  |
| 116. exp Human Rights/ |  |
| 117. Legal services.mp. |  |
| 118. exp Vocational Education/ or exp Rehabilitation, Vocational/ or Vocational training.mp. |  |
| 119. Mentoring.mp. |  |
| 120. exp Community Mental Health Services/ or community oriented public mental health service*.mp. |  |
| 121. Resettlement assessment.mp. |  |
| 122. Outreach.mp. |  |
| 123. exp Self-Help Groups/ |  |
| 124. Psychotherapeutic intervention*.mp. |  |
| 125. Psychological first Aid.mp. |  |
| 126. ('implosive therap*' or flooding therap*' or 'imaginal floodings').mp. [mp=title, abstract, original title, name of substance word, subject heading word, keyword heading word, protocol supplementary concept word, rare disease supplementary concept word, unique identifier] |  |
| 127. 65 or 66 or 67 or 68 or 69 or 70 or 71 or 72 or 73 or 74 or 75 or 76 or 77 or 78 or 79 or 80 or 81 or 82 or 83 or 84 or 85 or 86 or 87 or 88 or 89 or 90 or 91 or 92 or 93 or 94 or 95 or 96 or 97 or 98 or 99 or 100 or 101 or 102 or 103 or 104 or 105 or 106 or 107 or 108 or 109 or 110 or 111 or 112 or 113 or 114 or 115 or 116 or 117 or 118 or 119 or 120 or 121 or 122 or 123 or 124 or 125 or 126 |  |
| 128. 24 and 64 and 127 |  |
| 129. limit 128 to (english language and humans and yr="1980 -Current") |  |
| 130. Ethnography.mp. |  |
| 131. Content analysis.mp. |  |
| 132. Participant observation.mp. |  |
| 133. Field note*.mp. |  |
| 134. exp "Process Assessment (Health Care)"/ or Process evaluation.mp. or exp "Outcome and Process Assessment (Health Care)"/ |  |
| 135. (Process measure* or process assessment*).mp. [mp=title, abstract, original title, name of substance word, subject heading word, keyword heading word, protocol supplementary concept word, rare disease supplementary concept word, unique identifier] |  |
| 136. Ethnopsychology.mp. or exp Ethnopsychology/ |  |
| 137. exp Qualitative Research/ or exp Focus Groups/ or Focus group*.mp. |  |
| 138. (Qualitative method* or Qualitative stud*).mp. [mp=title, abstract, original title, name of substance word, subject heading word, keyword heading word, protocol supplementary concept word, rare disease supplementary concept word, unique identifier] |  |
| 139. (group interview* or in-depth interview* or one-to-one interview*).mp. [mp=title, abstract, original title, name of substance word, subject heading word, keyword heading word, protocol supplementary concept word, rare disease supplementary concept word, unique identifier] |  |
| 140. Mixed-methods.mp. |  |
| 141. Thematic synthesis.mp. |  |
| 142. thematic analysis.mp. |  |
| 143. qualitative analysis.mp. |  |
| 144. framework synthesis.mp. |  |
| 145. framework analysis.mp. |  |
| 146. Grounded theory.mp. or exp Grounded Theory/ |  |
| 147. (Grounded research or grounded stud*).mp. [mp=title, abstract, original title, name of substance word, subject heading word, keyword heading word, protocol supplementary concept word, rare disease supplementary concept word, unique identifier] |  |
| 148. Constant comparative.mp. |  |
| 149. Theoretical saturation.mp. |  |
| 150. realist.mp. |  |
| 151. Constructionist.mp. |  |
| 152. (Pragmatism or realism).mp. [mp=title, abstract, original title, name of substance word, subject heading word, keyword heading word, protocol supplementary concept word, rare disease supplementary concept word, unique identifier] |  |
| 153. exp Feminism/ or Feminis*.mp. |  |
| 154. Social construction.mp. |  |
| 155. stakeholder view*.mp. |  |
| 156. acceptability.mp. |  |
| 157. affordability.mp. |  |
| 158. accessibility.mp. |  |
| 159. Implementation science.mp. |  |
| 160. exp Community-Based Participatory Research/ or Participatory research.mp. |  |
| 161. Intervention delivery.mp. |  |
| 162. fidelity.mp. |  |
| 163. Needs assessment.mp. or exp Needs Assessment/ |  |
| 164. 130 or 131 or 132 or 133 or 134 or 135 or 136 or 137 or 138 or 139 or 140 or 141 or 142 or 143 or 144 or 145 or 146 or 147 or 148 or 149 or 150 or 151 or 152 or 153 or 154 or 155 or 156 or 157 or 158 or 159 or 160 or 161 or 162 or 163 |  |
| 165. 64 and 127 and 164 |  |
| 166. limit 165 to (english language and humans and yr="1980 -Current") |  |
| 167. 129 or 166  **Websites searched**   - The World Bank: <http://www.worldbank.org/> - The Overseas Development Institute (ODI), including the Humanitarian Policy Group: <http://www.odi.org/programmes/humanitarian-policy-group> (HPG) and Humanitarian Practice Network: <http://odihpn.org/> (HPN) - Institute of Development Studies: <http://www.ids.ac.uk/> - International Development Research Centre: <http://www.idrc.ca/EN/Pages/default.aspx> - Active Learning Network for Accountability and Performance in Humanitarian Action (ALNAP): <http://www.alnap.org/> - Emergency Nutrition Network (Field Exchange): <http://www.ennonline.net/> - Evidence Aid: <http://www.evidenceaid.org/> - Feinstein International Center, Tufts University: <http://fic.tufts.edu/> - Enhanced Learning and Research for Humanitarian Assistance: <http://www.elrha.org/> - International Association of Professionals in Humanitarian Assistance and Protection: <https://phap.org/> - Humanitarian Accountability Partnership: <http://www.hapinternational.org/> (now CHS Alliance) - Network on Humanitarian Action: <http://nohanet.org/> - Harvard Humanitarian Initiative: <http://hhi.harvard.edu/> - Refugee Studies Centre, University of Oxford: <http://www.rsc.ox.ac.uk/> - European Commission Humanitarian Aid and Civil Protection Department (ECHO): <http://ec.europa.eu/echo/> - USAID Development Experience Clearinghouse (and related USAID sub-websites): <https://dec.usaid.gov/dec/home/Default.aspx> - ReliefWeb: <http://reliefweb.int/> - Oxfam Policy and Practice: <http://policy-practice.oxfam.org.uk/> - Mental Health and Psychosocial Support Network: <http://mhpss.net/> - UNHCR: <http://www.unhcr.org/cgi-bin/texis/vtx/home> - UNICEF: <http://www.unicef.org.uk/> - Asian Development Bank: <http://www.adb.org/about/main> - African Development Bank: <http://www.afdb.org/en/> - Inter-American Development Bank: <http://www.iadb.org/en/inter-american-development-bank,2837.html> - United Nations Office for the Coordination of Humanitarian Affairs (OCHA): <http://www.unocha.org/hina> - International Committee of the Red Cross (ICRC): <https://www.icrc.org/en> - Office of U.S. Foreign Disaster Assistance (OFDA), USAID: <https://www.usaid.gov/who-we-are/organization/bureaus/bureau-democracy-conflict-and-humanitarian-assistance/office-us> |  |
