# Supplementary material for: The impact of mental health and psychosocial support programmes on children and young people’s mental health in the context of humanitarian emergencies in low- and middle-income countries: A systematic review and meta-analysis
Source: Glob Ment Health (Camb). 2024 Feb 12;11:e21. doi: 10.1017/gmh.2024.17 (PMC10988149; doi:10.1017/gmh.2024.17)
Supplement: Bangpan et al. supplementary material 2 — Bangpan et al. supplementary material [file S2054425124000177sup002.docx]

Eligibility criteria

| Inclusion criteria | Exclusion criteria |
| --- | --- |
| **Participants**: Children and young people (aged 0-25 years old) affected by humanitarian emergencies or those who have been exposed to events leading to MHPSS impacts as a result of a humanitarian crisis.  Humanitarian emergencies refer to natural and/or manmade disasters, including slow-onset and sudden crises. These included, but were not limited to, earthquakes, volcanoes, rock falls, avalanches, landslides, storms, hurricanes, floods, extreme temperatures, wildfires, terrorist attacks, political violence, and armed conflicts. | We exclude studies where exclusively focused on:  Adults older than 25 years old. We excluded studies that children and young people whose average age was older than 25 years old.  Military personnel working in the context of humanitarian emergencies |
| **Intervention**: Programmes which seek to provide MHPSS interventions delivered in the context of humanitarian emergencies or for populations affected by humanitarian emergencies in low- and middle-income countries (LMICs) | We exclude studies:  Delivering MHPSS interventions not in the context of humanitarian emergencies or not to children and young people affected by humanitarian emergencies in LMICs. |
| **Study design:**  Prospective experimental and quasi-experimental studies including randomized controlled trials and non-randomized controlled trials with control groups. Comparison groups can be those with no intervention, other active interventions, or usual care. | We exclude studies that did not:  report an outcome evaluation of MHPSS interventions using prospective experimental and quasi-experimental studies with control groups. |
| **Reported data:**  We include any type of mental health or psychosocial outcomes reported in the study (e.g. individual, family, community, social outcomes). | We exclude studies that did not collect and report outcome data on the impact of an MHPSS programme |
| **Language**: Published in English. | We exclude studies:  Not published in English |
| **Date:** Published in or after 1980. | Published before 1980 |
