# Supplementary material for: The impact of mental health and psychosocial support programmes on children and young people’s mental health in the context of humanitarian emergencies in low- and middle-income countries: A systematic review and meta-analysis
Source: Glob Ment Health (Camb). 2024 Feb 12;11:e21. doi: 10.1017/gmh.2024.17 (PMC10988149; doi:10.1017/gmh.2024.17)
Supplement: Bangpan et al. supplementary material 3 — Bangpan et al. supplementary material [file S2054425124000177sup003.docx]

Characteristics of 60 studies

| Study | Setting | Population | Intervention | Format | Delivery setting | Dose/ Intensity/ duration | Person who delivered |
| --- | --- | --- | --- | --- | --- | --- | --- |
| Ager (2011) | Uganda  Armed conflict | Primary school students; age 7-12 years; mean age =10.23 (1.61)  Female =50.1% | School-based Psychosocial Structured Activities: involving drama, movement, music, and art | Group | Classroom | 15, 60 mins sessions, over 5 weeks | Teachers |
| Akiyama (2011) | The Philippines,  Typhoon 2013 | 10th grade students; mean age 16.6 (1.61)  Female = 56.66% | The Mastery Approach to Coaching (MAC): a coaching-education programme involving sports activity | Group | School and outdoor | 1 hour per day for 4 days a week, over two months | Teachers |
| Ahmadi (2023) | Afghanistan  Armed conflict | 125 participants: mean (SD) age of 15.96 (1.97) years | the Memory Training for Recovery-Adolescent Intervention | Group | Private room | 10, 1 hour sessions over 2 weeks | Community leaders/workers |
| Annan (2017) | Thailand  Armed conflict | Burmese migrant and displaced children  Female = 51% | Parent and Family Skills interventions adapted from the Strengthening Families Programme (SFP) originally developed in USA for substance abusing parents and their children | Group | Community spaces such as school and community halls | 12 weekly, 2-hrs, sessions | Local lay facilitators |
| Barron (2013) | Palestine  Armed conflict | Public school students; age 11-13 yeas; mean age = 11.09 (1.19); Female = 42.14%; | The Arabic Teaching Recovery Techniques (TRT) -A Cognitive Behavioural Therapy (CBT) programme that focuses on normalizing trauma and strategies for intrusive memories, hyper-arousal, and avoidance symptoms of PTSD | Group | School | 5 weekly, 90 mins, sessions, over 5 weeks | School Counsellors |
| Barron (2016) | Palestine  Armed conflict and political violence | School students; age 11-15 years; mean age = 13.57 (0.82)  Female= 59.7% | The Teaching Recovery Techniques (TRT). The group-delivered program, based on CBT, focuses specifically on children’s symptoms of PTSD. | Group | School | 5 weekly, 90 mins, session, over 5 weeks | Counsellors |
| Berger (2009) | Sri Lanka  Tsunami | Elementary school students; age 9-14 years  Female = 41.7% | ERASE Stress Sri-Lanka (ES-SL)- a classroom-based programme designed to help children cope with the threat and the exposure to disaster and trauma. The programme involved children’s caregivers with home assignments | Group | School- classroom | 12, 90 min sessions | Teachers |
| Berger (2018) | Tanzania    Armed conflict | School children, Mean age: INT = 12.44 (0.89); CON = 88, mean age = 12.48 (0.93); Females = 50.8% | Erase Stress Prosocial (ESPS) is a universal school-based program divided into two sets of strategies: stress-reduction interventions and pro-social interventions (i.e. perspective-taking, empathy training, mindfulness and  compassion-cultivating practices). | Group | School | 16 sessions, 90 mins weekly | Teachers |
| Betancourt (2014) | Sierra Leone  Armed conflict | Youth; mean age =18 years; Female 45.6% | The Youth Readiness Intervention combines elements drawn primarily from CBT and Interpersonal therapy, both evidence-based therapies with  demonstrated effectiveness in treating depression, anxiety, and interpersonal deﬁcits due to trauma. | Group | Community sites | 10-12, 90 min sessions, over 10 weeks | Local lay counsellors |
| Bolton (2007) | Uganda  Armed conflict | Acholi adolescent age 14-17 years from 2 IDP camps: Female 57.3% | Group Interpersonal Psychotherapy,  Creative play- aiming to strenthening youth’s resilience by verbal and nonverbal expression of thoughts and feelings through activities such as songs, art, role plays, music, sports, games | Group | Camps | 16, 90-120 min, sessions | Facilitators |
| Brown (2009) | Rwanda  Genocide and AIDS Pandemic | Youth heads of household, ages between 12-24 years; Gender: Female 47% | Adult mentorship and support to improve psychosocial outcomes among youth-headed households in a rural area of Rwanda | Individual | Home | at least twice a month, for 2-3 hours, over 18 months | volunteer mentors from community |
| Brown (2023) | Lebanon  Armed conflict | 67 children age 10-14; Forty-45% female; average age was 11.7 (SD = 1.3). | Early Adolescent Skills for Emotions (EASE) | Group | Community | 7 weekly 1.5 hours sessions | Local facilitators |
| Bryant (2022) | Jordan  Armed conflict, refugees | 471 Syrian refugees aged 10 to 14 years | Early Adolescent Skills for Emotions’; EASE | Group | Community | 7 weekly 1.5 hours sessions | Local facilitators |
| Catani  (2009) | Sri Lanka  Tsunami and war | Children aged 8-14 years, Female 45.16% | KIDNET- the participants constructed a detailed chronological account of his or her own biography | Individual | Refugee camps | 6, 60-90 min, sessions over two weeks | Local teacher counsellors |
| Chen (2014) | China  Earthquake | Adolescents from two secondary schools; mean age = 14.50 y; female 68% | Cognitive Behavioural Therapy (CBT) | Group | INT 1 =not stated,  INT2: home | 6, 60 min, sessions, weekly | School staff |
| Cleodora (2018) | Indonesia Earthquake and Tsunami | 69 children: INT = 35, mean age = 10.69; CONT = 34, mean age =10.59 | Therapeutic Group Therapy (TGT- focusing on the protective fact of self-efficacy and is conducted through giving simulation on developmental according to age | Group | Not stated | 7, 1-1.5 hours, sessions | Not stated |
| Cluver (2015) | Haiti  Earthquake | Children living in orphanages; mean age = 11.23 y; Female 42% | Yoga | Group | School room | 8 weeks, twice, 45 mins, weekly | Not stated |
| Dawson (2018) | Indonesia  Civil war | Children aged between 7-14; mean age: INT = 10.50 (0.93);  CON = 10.25 (1.16) | Trauma-focused CBT with children and caregivers | Individual | Not stated | 1, 1 hour session over 5 weeks | Counsellors |
| Dhital (2019) | Nepal  natural disaster | Students grade 6-8; mean age INT = 12.9 (1.3); CON = 12.9 (1.4) | Psychosocial: Teacher-mediated school-based intervention | Group | School | 2 days training, eight sessions, 1-2 hours | Teachers |
| Dybdahl (2001) | Bosnia and Herzegovina  Armed conflict | Bosnian-displaced mothers; Female only (Mothers)- Child (Mean age = 5.5 y; Female =55.17%) | Psychosocial intervention aiming to promote the development and well-being of young children through parental involvement, support and education, and parent-child relationships | Group | Not stated for the main intervention component, home visit | Weekly group meeting for five months; 60 min, home visit | School teachers for interventions also physician for medication group |
| El-Khani (2021) | Lebanon  Armed Conflict | Children and their caregivers: Child age 9-12 years; | Child Trauma recovery programme Teaching Recovery Techniques (TRT) with parent sessions | Group | Unclear | 5 weekly sessions, 120 mins. | Teachers |
| Ertl (2011) | Uganda  Civil war | Child soldiers;  Female 67.1% | Narrative Exposure Therapy (NET) -a short term, trauma-focused treatment | Individual | IDP camp | 8 sessions, 90-120 mins, 3 times a week | Local lay counsellors |
| Fine (2021) | Tanzania  Armed conflict | Young adolescents and their caregivers living in refugee camps  Female 49%; mean age = 12.3(1.5) | The Early Adolescent Skills for Emotions (EASE) intervention aiming to reduce symptoms of internalising disorders, including depression and anxiety. | Group | Refugee camp | 7 weekly, 90 mins, sessions | Local lay volunteers |
| Geonjian (2005) | Armenia  Earthquake | Children  INT =35 and CON =29 | The psychiatric outreach programme, a trauma/grief-focused treatment was delivered during school hours | Group and Individual | School Classroom | 4, 30 mins, sessions. over three weeks | Skilled mental health professional from USA |
| Getanda and Vostanis (2020) | Kenya  Displacement | Youth aged 14-17 years  N =54 | Writing for Recovery- focusing on sensory aspects of traumatic events leading to PTSD and other symptoms of distress | Group | School | 6 sessions over 3 days | Local facilitators |
| Gordon (2008) | Kosovo  Armed conflict | Children; mean age = 16.3 y; Female = 75.60% | Mind-body technique model- a combination of a number of mind-body modalities with self-expression (spoken, written words, drawings, movement) | Group | School | 12, 120 min, session, twice weekly for 6 weeks | Teachers |
| Hasanovic (2009) | Bosnia-Herzegovina  Armed conflict | Students in primary and secondary schools in intervention; aged 12-15 years; gender mixed, mean aged 13.5(1.6) | The school project of 'supporting returning students to integrate into the school system in post war Bosnia and Herzegovina’. The programme also worked with parents, school management and teachers. | Group | School | 20 lessons for 5 months; and other components, workshops | Researchers |
| Jordans (2010) | Nepal  Armed conflict | School children; mean age = 12.7 years; girls 48.6% | A school-based psychosocial intervention- the Classroom-Based Intervention (CBI)- an eclectic intervention based on concepts from creative-expressive and experimental therapy, cooperative play and CBT | Group | Classroom | 15, 60 min, sessions over 5 weeks | Research assistants |
| Jordans (2013) | Burandi  Civil War | School children, mean age 12.3 years (SD = 1.51); girls = 60.8% | The brief psychoeducation intervention including working with parents | Group | Not stated | 2 sessions, 2.5- 3 hours, over 3 months | Lay community counsellors |
| Kalantari (2012) | Iran  Armed conflict | Afghan refugees in school; mean age INT = 14.58 y; CON = 15.03 y; Females 55% | Writing For Recovery – the writing sessions developed for adolescents who have experienced a trauma. | Group | School | 3 consecutive days: two, 15 mins, sessions, a day | Not stated |
| Karam (2008) | Lebanon  Armed conflict | Students grades 1-9 from 6 schools; mean age 11.7(2.7) in intervention and 11. (3.1) in control group | The intervention components is a combination of cognitive-behavioural strategies and stress inoculation training | Group | School | 12, 1 hr sesions, daily over 12 days | Teachers |
| Khamis (2004) | Palestine  Armed conflict | Children aged 6-16 years, Female 43.37% | CBT: Classroom-based intervention-a psychosocial integration and recovery programme for children and adolescents and their adult caregivers who are exposed to psychological trauma | Group | School or camp | 15 sessions over 5 weeks | Social workers, school counsellors, other psychological support personnel |
| Lange-Nelsen (2012) | Gaza  Armed conflict | 124 Adolescents; mean age = 14.54 years; Female 50% | Writing For Recovery-a manual-based group intervention aimed at adolescents who have a history of trauma through short writing sessions | Group | School | 2, 15 min, sessions per day for 3 days | Psychologists |
| Layne (2008) | Bosnia  Armed conflict | War exposed secondary school students from 10 schools; Muslim; Age ranging from 14-19 years, mean age = 16(1.13) | A manual based psychotherapy and a classroom-based psycho education programme. | Group | School | 17-20 weekly group sessions for 7 months (school year), between 60 to 90mins | School Counsellors (psychologists, pedagogues) |
| Lesmana (2009) | Indonesia  Terrorist attack | Children (Female 52.7%) mean age =9.83 (1.53) | A spiritual-hypnosis assisted therapy | Group | Not stated | One single session, about 30 minutes | Researcher |
| Loughry (2006) | Palestine  Armed conflict | 400 children from the West Bank (n=250) and Gaza (n= 150); aged between 6-17 years old; Gender, mixed | A child-focused interventions involving structured activities including participation in recreational, cultural and other non-formal activities. | Group | Community local recreation centres | Not stated | Local young adult volunteers |
| McMullen (2013) | Congo  Armed conflict | 39 former soldiers and 11 war-affected boys; mean age = 15.8 years | A manualised, Trauma-focused CBT | Group | School | 15 sessions | Researchers and counsellors |
| Morris (2012) | Uganda  Armed conflict | Internally displaced mothers and babies for mothers attending emergency feeding centres | A group-based psychosocial intervention delivered to mothers and babies and home visits | Group and home visits | Community-based | 90-120 mins for mother-child sessions | Trained local psychosocial facilitators |
| Nopembri (2019) | Indonesia  Natural disaster | Students: INT = mean age 10.4-10.39 y ears | Physical Education and sports classes | Group | School | 15, 2 hr sessions, 3 days a week over five weeks | Social workers |
| O’Callaghan (2013) | Congo  Armed conflict | Girls who had witnessed or had personal experience of rape of sexual abuse; mean age =16 years | A manualised, culturally modified, trauma focused CBT | Group | School | 15, 120 mins, sessions, 3 days a week over five weeks | Social workers |
| O’Callaghan (2014) | Uganda  Armed conflict | Children: mean age = 13.42 years; females 45% | A manualised, family-focused- psychosocial interventions | Group | Church | 3 times weekly, 8, 12o mins, sessions over 4 weeks | Local lay facilitators |
| O’Callaghan (2015) | Congo  Armed conflicted | War affected youth; mean age = 14.88 years | Trauma-focused CBT- a combination of cognitive therapy and behavioural therapy  Child Friendly Spaces- a psychosocial intervention aiming to improve resilience and wellbeing of youth through community-based, structured activities held in a safe, child friendly environment | Individual | Field attached to local schools | 9, 90 mins, sessions, three sessions per week plus two, 90 mins caregivers’ sessions in group sessions | Local teachers as a facilitator and social workers |
| Panter-Brick (2018) | Syria  Armed conflict | Adolescents, mean age = 14.37 (1.72), Female = 43% | Advancing Adolescents programme- consisting of structural activities that aimed to promote capacities for the mediation of extreme and prolonged stress | Group | community based (Youth centres, designed as ‘Adolescent Friendly Spaces) | 16 sessions (twice per week) for 8 weeks | Local facilitators/volunteers |
| Peltonen (2012) | Palestine  Conflict | Palestinian school children | School Mediation Intervention | Group | School | Part of school year | Students, teachers as supervisors |
| Pityaratstian (2015) | Thailand  Tsunami | Children; mean age =12.25 years: Female = 72.2% | CBT- a manual-based and adapted from the Teaching Recovery Techniques (TRT) | Group | School and outdoor | Daily, 120 mins, sessions for 3 days | Psychiatrists |
| Quata (2012) | Palestine  Armed conflict | Children; mean age = 11.29 years; Female 49.4% | Extracurriculum sessions of the Teaching recovering techniques (TRT) | Group | School | 2, weekly, 2hr session in total of 16 sessions, last for 4 weeks | Psychologists |
| Richards (2014) | Uganda  Armed conflict | Children; mean age = 9.83 years | Voluntary competitive sports for development football league | Group | Sport field | 11 weeks – 45 mins per session | Local volunteers |
| Robjant (2019) | Democratic Republic of Congo  Armed conflict | 92 female former child soldiers, age range 11-25 years | Narrative Exposure Therapy adapted for offenders (FORNET) | group and individual | Community based | 6 individual sessions, 90-120 mins; group therapy weekly for 60-90 mins | Lay therapists |
| Schaal (2009) | Rwanda  Genocide | Children mean aged 19.42 (3.59) range 14-28 years | NET involving guided mourning | Individual | Not stated | 4 weekly sessions about 120-150 mins | Female counsellors |
| Schauer (2008) | Sri Lanka  Armed conflict and Tsunami | Children who suffered severe PTSD; mean age = 13.1 years | Manualised KIDNET- a trauma-focused, short term therapy with exposure elements | Group | School | 6, 60-90 mins, sessions | Teacher Counsellors |
| Shoaakazemi (2012) | Iran  Earthquake | Girls with PTSD (15-18 years) | Psychotherapy: Logo therapy | Individual | Not stated | 8 ,60 mins, sessions | Health professionals |
| Shooshatary  (2008) | Iran  Earthquake | Adolescents; Mean age = 15.50(2.3) years | CBT | Group | Not stated | 4, 2 hr sessions over 4 weeks | Residents of psychiatry |
| Sirin (2018) | Turkey  Conflict | Syrian refugees age range 9-14; mean age 11.75 (1.23) years | Project Hope: an online-game based learning intervention | Group | Community based | 2 hours, 5 days a week for 4 weeks | Teachers |
| Thabet (2005) | Palestine  Armed conflict | Children from five refugee camps | Crisis intervention- adjusted for trauma by using free drawing, talking about experiences and feelings, writing, storytelling, games, role plays  Teacher education | Group | Refugee camps | 7, weekly sessions | Psychiatrist, psychologists and social workers |
| Tol (2008) | Indonesia  Political violence | School children, mean age = 9.9 years, Girls 48.63% | School-based mental health including trauma-processing activities, cooperative play, and creative expressive elements | Group | Classroom | 15 sessions, over 5 weeks | Local community workers |
| Tol (2012) | Sri Lanka  Armed conflict | Children; mean age 12.29 years; Girls 48% | School-based mental health- consisting of CBT and creative expressive elements | Group | School | 15 sessions for 5 weeks | Local, trained, non-specialised staff |
| Tol (2014) | Burundi  Armed conflict | Children, mean age 12.29 years | The classroom-based intervention (CBI) including universal preventive activities and provision of mental health treatments | Group | School | 15 sessions for 5 weeks | Local facilitators |
| Torrente (2019) | DRC  Armed conflict | 76 clusters and 126 schools in total of 8813 students, 47.5%  females, mean age = 11.66 (1.9) years | Learning in a Healing Classroom- a universal school-based intervention to support children’s social-emotional well-being and academic learning | Group | School | One to two academic years | Teachers and teaching staff |
| Veronese (2018) | Palestine  Conflict | 64 children age range 8-14 years; Mean age INT = 11.33 (0.68) years; CONT= 11.68 (0.63) years | An expressive- narrative psychosocial intervention | Group | School | 6 sessions, 4 hours over a weeklong period | local social workers, teachers and Therapist |
| Yankey (2019) | India  Political violence, refugees | Tibetan refugees, age 13-17 years | School-based life skill training programme | Group | School | 30 sessions over 11 months | Not stated |

*INT = Intervention; CON = comparison group; IDP = Internally displaced person
