# Supplementary material for: The impact of mental health and psychosocial support programmes on children and young people’s mental health in the context of humanitarian emergencies in low- and middle-income countries: A systematic review and meta-analysis
Source: Glob Ment Health (Camb). 2024 Feb 12;11:e21. doi: 10.1017/gmh.2024.17 (PMC10988149; doi:10.1017/gmh.2024.17)
Supplement: Bangpan et al. supplementary material 4 — Bangpan et al. supplementary material [file S2054425124000177sup004.docx]

Forest plots

1. The impact of MHPSS on PTSD


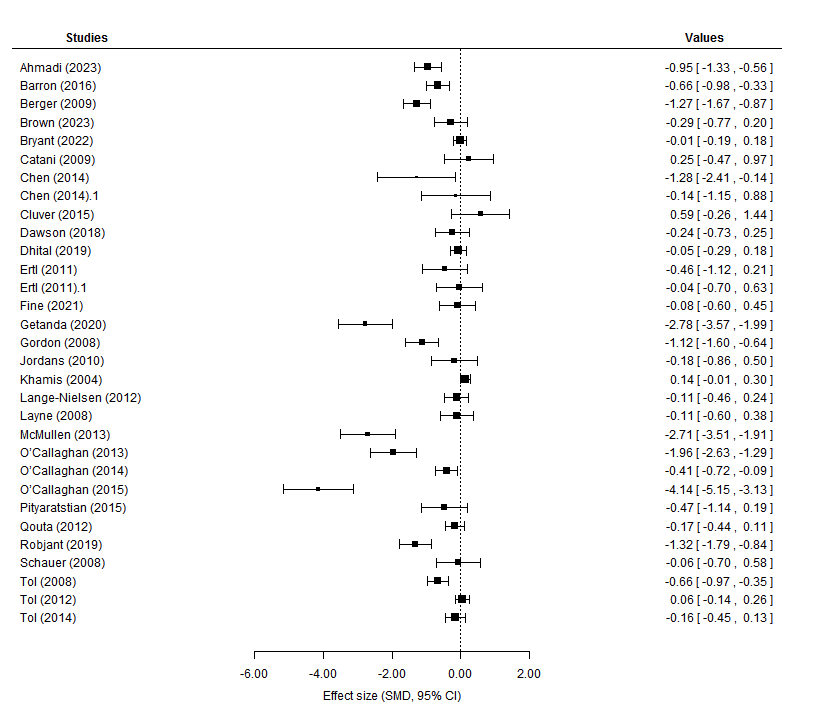


1. The impact of MHPSS on depression


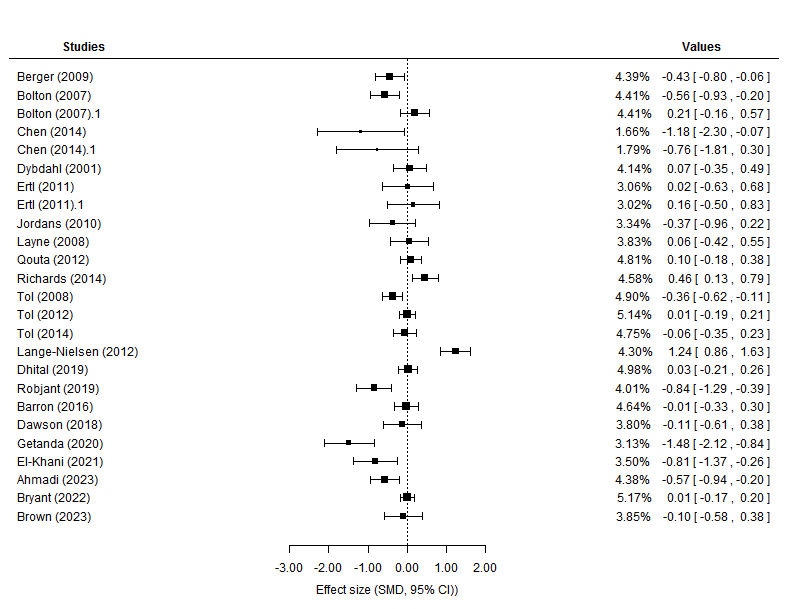


1. The impact of MHPSS on conduct problems


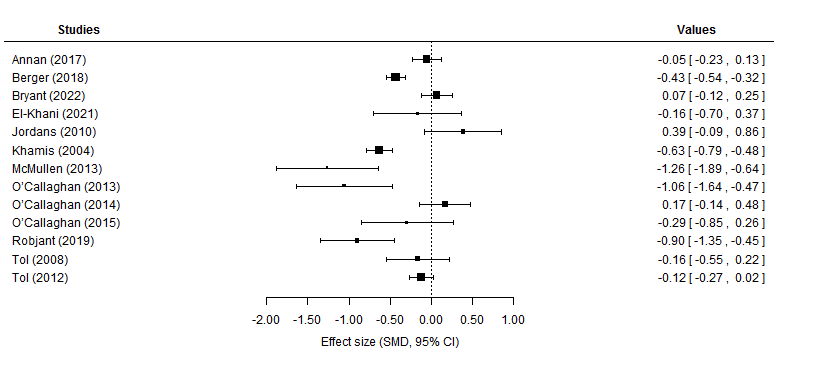


1. The impact of MHPSS on functional impairment


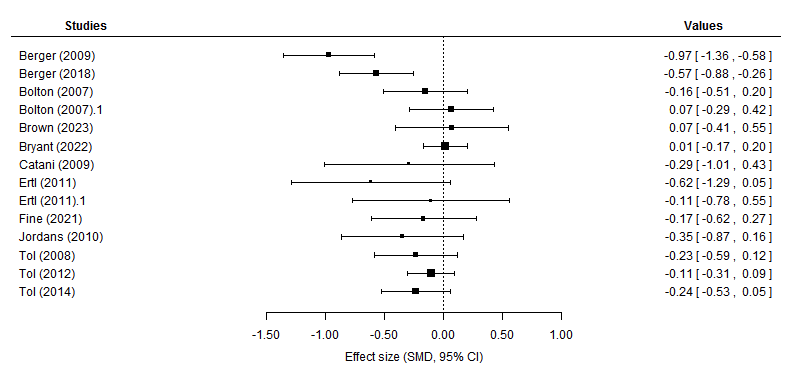


1. The impact of MHPSS on prosocial behaviour


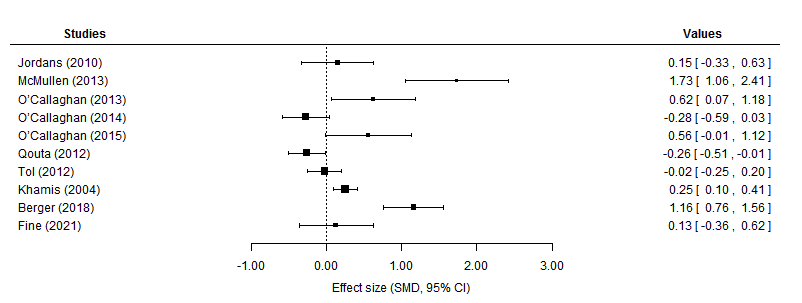


1. The impact of MHPSS on anxiety


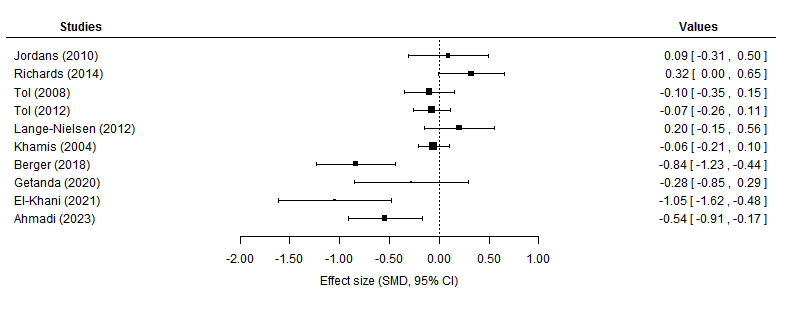


1. The impact of MHPSS on psychosocial distress


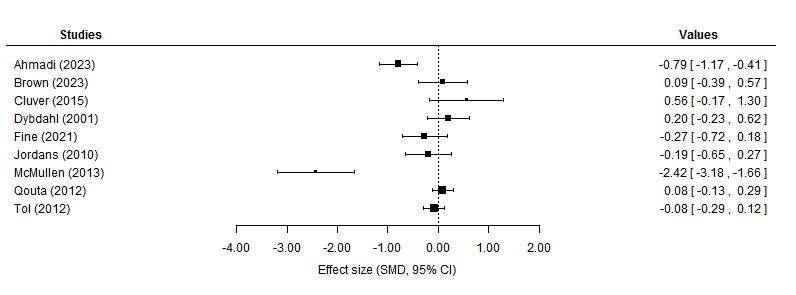


1. The impact of MHPSS on emotional problems


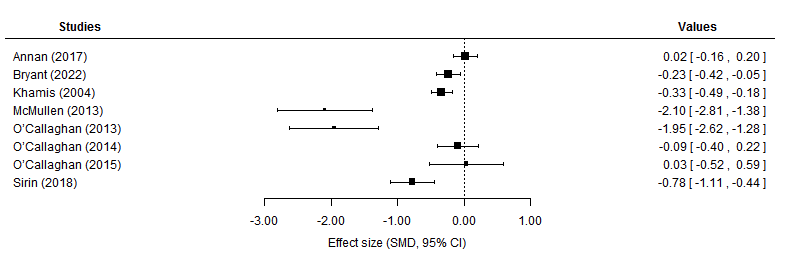


1. The impact of MHPSS on hope


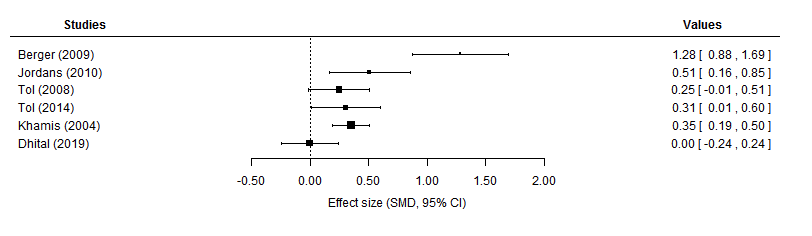


1. The impact of MHPSS on somatic complaints


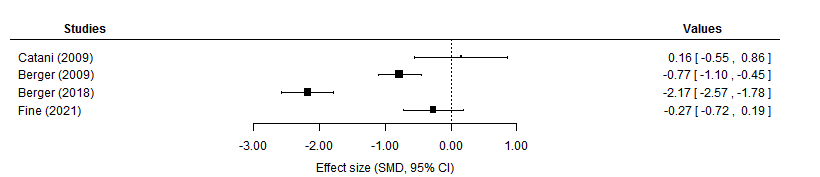


1. The impact of MHPSS on coping


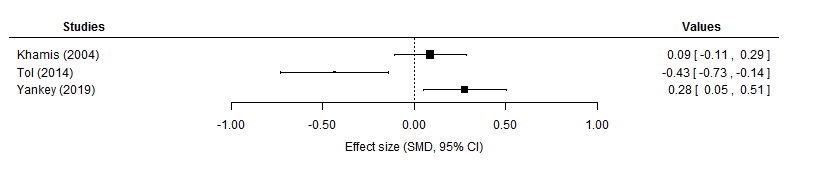


1. The impact of MHPSS on grief


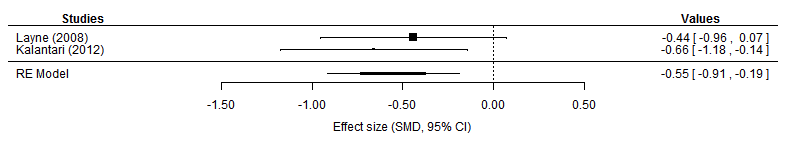


#### Main Summary

Random-Effects Model (k = 2; tau^2 estimator: DL)

tau^2 (estimated amount of total heterogeneity): 0 (SE = 0.10)
tau (square root of estimated tau^2 value): 0
I^2 (total heterogeneity / total variability): 0.00%
H^2 (total variability / sampling variability): 1.00

Test for Heterogeneity:
Q(df = 1) = 0.34, p-val = 0.56

Model Results:

estimate se zval pval ci.lb ci.ub
 -0.55 0.19 -2.97 <.01 -0.91 -0.19 **

1. The impact of MHPSS on guilt


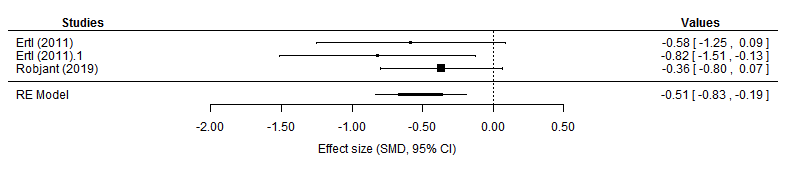


#### Main Summary

Random-Effects Model (k = 3; tau^2 estimator: DL)

tau^2 (estimated amount of total heterogeneity): 0 (SE = 0.09)
tau (square root of estimated tau^2 value): 0
I^2 (total heterogeneity / total variability): 0.00%
H^2 (total variability / sampling variability): 1.00

Test for Heterogeneity:
Q(df = 2) = 1.24, p-val = 0.54

Model Results:

estimate se zval pval ci.lb ci.ub
 -0.51 0.16 -3.12 <.01 -0.83 -0.19 **

1. The impact of psychosocial programmes on PTSD


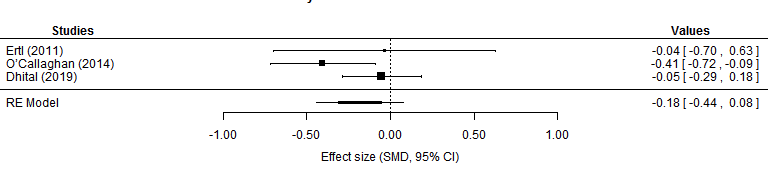


**Main Summary**

Random-Effects Model (k = 3; tau^2 estimator: DL)

tau^2 (estimated amount of total heterogeneity): 0.02 (SE = 0.05)
tau (square root of estimated tau^2 value): 0.14
I^2 (total heterogeneity / total variability): 39.30%
H^2 (total variability / sampling variability): 1.65

Test for Heterogeneity:
Q(df = 2) = 3.30, p-val = 0.19

Model Results:

estimate se zval pval ci.lb ci.ub
 -0.18 0.13 -1.38 0.17 -0.44 0.08

1. The impact of psychosocial programmes on conduct problems


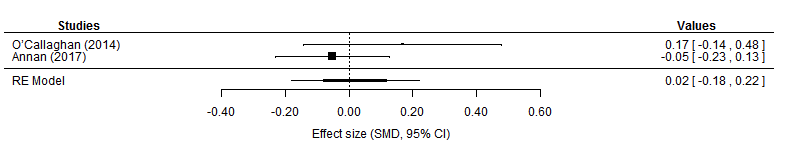


#### Main Summary

Random-Effects Model (k = 2; tau^2 estimator: DL)

tau^2 (estimated amount of total heterogeneity): 0.01 (SE = 0.03)
tau (square root of estimated tau^2 value): 0.08
I^2 (total heterogeneity / total variability): 30.03%
H^2 (total variability / sampling variability): 1.43

Test for Heterogeneity:
Q(df = 1) = 1.43, p-val = 0.23

Model Results:

estimate se zval pval ci.lb ci.ub
 0.02 0.10 0.19 0.85 -0.18 0.22

1. The impact of psychosocial programmes on emotional problems


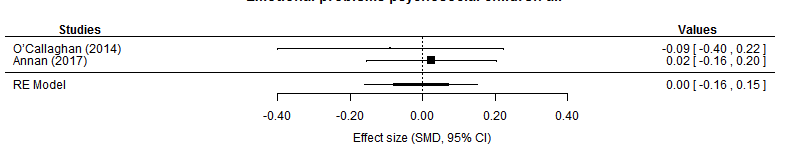


#### Main Summary

Random-Effects Model (k = 2; tau^2 estimator: DL)

tau^2 (estimated amount of total heterogeneity): 0 (SE = 0.02)
tau (square root of estimated tau^2 value): 0
I^2 (total heterogeneity / total variability): 0.00%
H^2 (total variability / sampling variability): 1.00

Test for Heterogeneity:
Q(df = 1) = 0.38, p-val = 0.54

Model Results:

estimate se zval pval ci.lb ci.ub
 -0.00 0.08 -0.05 0.96 -0.16 0.15

1. The impact of psychosocial programmes on functioning


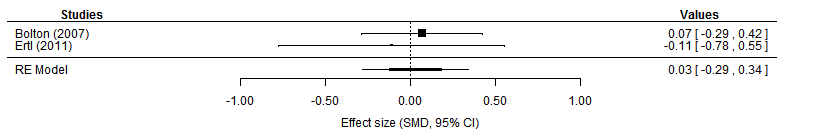


#### Main Summary

Random-Effects Model (k = 2; tau^2 estimator: DL)

tau^2 (estimated amount of total heterogeneity): 0 (SE = 0.10)
tau (square root of estimated tau^2 value): 0
I^2 (total heterogeneity / total variability): 0.00%
H^2 (total variability / sampling variability): 1.00

Test for Heterogeneity:
Q(df = 1) = 0.22, p-val = 0.64

Model Results:

estimate se zval pval ci.lb ci.ub
 0.03 0.16 0.18 0.86 -0.29 0.34

1. The impact of psychosocial programmes on depression


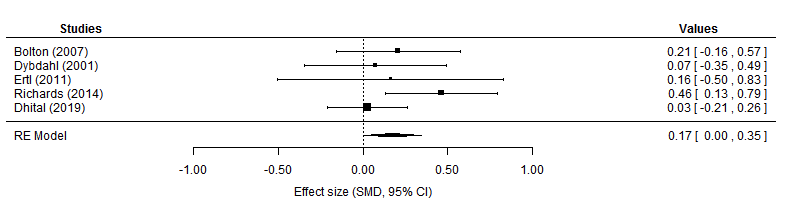


#### Main Summary

Random-Effects Model (k = 5; tau^2 estimator: DL)

tau^2 (estimated amount of total heterogeneity): 0.01 (SE = 0.03)
tau (square root of estimated tau^2 value): 0.08
I^2 (total heterogeneity / total variability): 14.49%
H^2 (total variability / sampling variability): 1.17

Test for Heterogeneity:
Q(df = 4) = 4.68, p-val = 0.32

Model Results:

estimate se zval pval ci.lb ci.ub
 0.17 0.09 1.99 0.05 0.00 0.35 *

1. The impact of NET on guilt


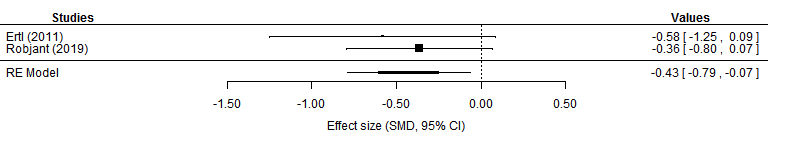


#### Main Summary

Random-Effects Model (k = 2; tau^2 estimator: DL)

tau^2 (estimated amount of total heterogeneity): 0 (SE = 0.12)
tau (square root of estimated tau^2 value): 0
I^2 (total heterogeneity / total variability): 0.00%
H^2 (total variability / sampling variability): 1.00

Test for Heterogeneity:
Q(df = 1) = 0.29, p-val = 0.59

Model Results:

estimate se zval pval ci.lb ci.ub
 -0.43 0.18 -2.32 0.02 -0.79 -0.07 *
