# Supplementary material for: The impact of mental health and psychosocial support programmes on children and young people’s mental health in the context of humanitarian emergencies in low- and middle-income countries: A systematic review and meta-analysis
Source: Glob Ment Health (Camb). 2024 Feb 12;11:e21. doi: 10.1017/gmh.2024.17 (PMC10988149; doi:10.1017/gmh.2024.17)

Risk of Bias tables

a) Risk of bias of individual RCTs

| **Short Title** | **Risk of bias arising from the randomisation process** | **Risk of bias due to deviations from the intended interventions** | **Missing outcome data** | **Risk of bias in measurement of the outcome** | **Risk of bias in selection of the reported results** | **Overall risk of bias** |
| --- | --- | --- | --- | --- | --- | --- |
| Ahmadi (2023) | + | + | - | + | + | - |
| Annan (2017) | ? | + | + | + | + | ? |
| Barron (2016) | + | + | + | + | + | + |
| Betancourt (2014) | + | + | + | + | + | + |
| Bolton (2007) | + | + | + | + | + | + |
| Brown (2023) | + | ? | + | + | + | ? |
| Bryant (2022) | + | + | + | + | + | + |
| Catani (2009) | ? | + | + | + | + | ? |
| Chen (2014) | ? | - | - | - | + | - |
| Cluver (2015) | ? | - | - | - | + | - |
| Dawson (2018) | + | + | + | + | + | + |
| Dybdahl (2001) | ? | - | + | + | + | - |
| El-Khani (2021) | ? | + | + | + | + | ? |
| Ertl (2011) | ? | + | + | + | + | ? |
| Getanda and Vostanis (2020) | ? | ? | + | - | + | - |
| Gordon (2008) | - | + | + | - | + | - |
| Kalantari (2012) | ? | ? | - | - | + | - |
| Khamis (2004) | ? | - | - | + | + | - |
| Lange-Nielsen (2012) | ? | ? | + | - | + | - |
| Layne (2008) | - | - | - | - | + | - |
| McMullen (2013) | + | ? | + | + | + | ? |
| O’Callaghan (2013) | + | + | + | + | + | + |
| O’Callaghan (2014) | + | ? | + | + | + | ? |
| O’Callaghan (2015) | + | + | + | + | + | + |
| Panter‐Brick (2018) | + | + | + | + | + | + |
| Pityaratstian (2015) | ? | ? | + | + | + | ? |
| Richards (2014) | ? | + | ? | + | + | ? |
| Robjant (2019) | + | ? | + | + | + | ? |
| SHoaakazemi (2012) | ? | ? | - | - | - | - |
| Sirin (2018) | ? | - | - | - | ? | - |
| Yankey (2019) | ? | - | - | ? | + | - |


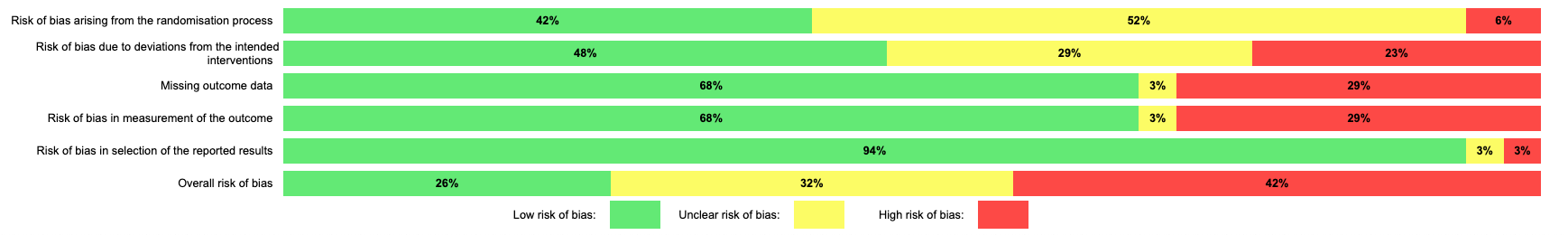


**B) Risk of bias of Cluster RCTs**

| **Short Title** | **Risk of bias judgement** | **Risk of bias arising from the timing of identification or recruitment of participants in a cluster-randomized trial** | **Risk of bias due to deviations from the intended interventions** | **Missing outcome data** | **Risk of bias in measurement of the outcome** | **Risk of bias in selection of the reported results** | **Overall risk of bias** |
| --- | --- | --- | --- | --- | --- | --- | --- |
| Berger (2009) | ? | + | ? | + | + | + | ? |
| Berger (2018) | + | + | + | - | + | ? | - |
| Dhital (2019) | + | ? | ? | - | - | + | - |
| Fine (2021) | + | ? | ? | + | + | ? | ? |
| Jordans (2010) | + | ? | + | + | ? | + | ? |
| Nopembri (2019) | - | + | + | - | - | + | - |
| Qouta (2012) | ? | + | + | + | ? | + | ? |
| Schauer (2008) | ? | + | + | + | + | + | ? |
| Tol (2008) | ? | ? | + | + | + | + | ? |
| Tol (2012) | ? | + | + | + | + | + | ? |
| Tol (2014) | - | - | ? | + | - | + | - |
| Torrente (2019) | + | + | + | - | + | + | - |


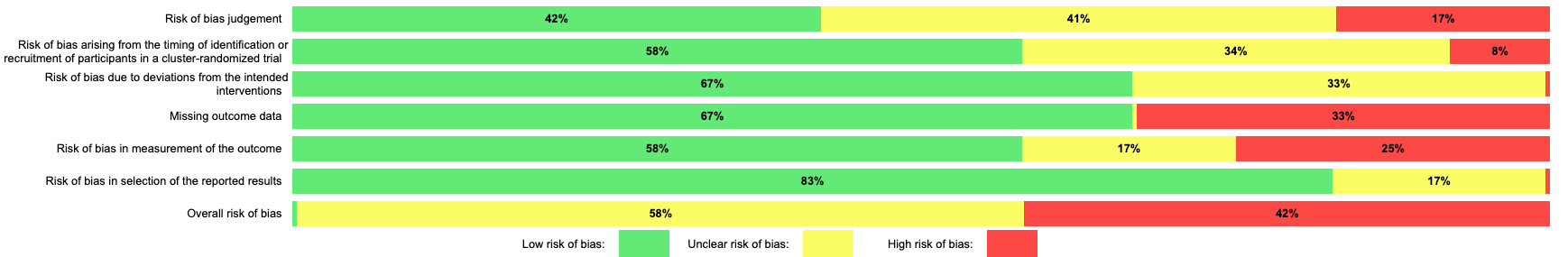

Supplement: Bangpan et al. supplementary material 5 — Bangpan et al. supplementary material [file S2054425124000177sup005.docx]
