# Supplementary material for: The impact of mental health and psychosocial support programmes on children and young people’s mental health in the context of humanitarian emergencies in low- and middle-income countries: A systematic review and meta-analysis
Source: Glob Ment Health (Camb). 2024 Feb 12;11:e21. doi: 10.1017/gmh.2024.17 (PMC10988149; doi:10.1017/gmh.2024.17)
Supplement: Peer review history

# Editor report: Recommendation: The impact of mental health and psychosocial support programmes on children and young people’s mental health in the context of humanitarian emergencies in low- and middle-income countries: A systematic review and meta-analysis — R0/PR2

**DOI:** 10.1017/gmh.2024.17.pr2

**Affiliations:** University of KwaZulu-Natal, South Africa

Kindly attend to the reviewers' comments and recommendations.

# Editor report: Decision: The impact of mental health and psychosocial support programmes on children and young people’s mental health in the context of humanitarian emergencies in low- and middle-income countries: A systematic review and meta-analysis — R0/PR3

**DOI:** 10.1017/gmh.2024.17.pr3

**Affiliations:** London School of Hygiene & Tropical Medicine, United Kingdom of Great Britain and Northern Ireland

No accompanying comment.

# Editor report: Recommendation: The impact of mental health and psychosocial support programmes on children and young people’s mental health in the context of humanitarian emergencies in low- and middle-income countries: A systematic review and meta-analysis — R1/PR5

**DOI:** 10.1017/gmh.2024.17.pr5

**Affiliations:** University of KwaZulu-Natal, South Africa

Thank you for the thorough revisions made. Kindly attend to the additional revision requests by the second reviewer. Kindly also review corrections made to address minor editorial mistakes that have arisen during the revision process.

# Editor report: Decision: The impact of mental health and psychosocial support programmes on children and young people’s mental health in the context of humanitarian emergencies in low- and middle-income countries: A systematic review and meta-analysis — R1/PR6

**DOI:** 10.1017/gmh.2024.17.pr6

**Affiliations:** London School of Hygiene & Tropical Medicine, United Kingdom of Great Britain and Northern Ireland

No accompanying comment.

# Editor report: Recommendation: The impact of mental health and psychosocial support programmes on children and young people’s mental health in the context of humanitarian emergencies in low- and middle-income countries: A systematic review and meta-analysis — R2/PR8

**DOI:** 10.1017/gmh.2024.17.pr8

**Affiliations:** University of KwaZulu-Natal, South Africa

No accompanying comment.

# Editor report: Decision: The impact of mental health and psychosocial support programmes on children and young people’s mental health in the context of humanitarian emergencies in low- and middle-income countries: A systematic review and meta-analysis — R2/PR9

**DOI:** 10.1017/gmh.2024.17.pr9

**Affiliations:** London School of Hygiene & Tropical Medicine, United Kingdom of Great Britain and Northern Ireland

No accompanying comment.
